# Surface-Initiated Polymerizations Mediated by Novel Germanium-Based Photoinitiators

**DOI:** 10.1021/acsami.3c05528

**Published:** 2023-06-23

**Authors:** Matthias Müller, Manfred Drusgala, Roland C. Fischer, Ana Torvisco, Wolfgang Kern, Michael Haas, Christine Bandl

**Affiliations:** † Montanuniversität Leoben, Institute of Chemistry of Polymeric Materials, Otto-Glöckel-Strasse 2, A-8700 Leoben, Austria; ‡ Graz University of Technology, Institute of Inorganic Chemistry, Stremayrgasse 9, A-8010 Graz, Austria; § Polymer Competence Center Leoben GmbH, Roseggerstrasse 12, A-8700 Leoben, Austria

**Keywords:** photopolymerization, surface modification, acylgermanes, photoinitiator immobilization, grafting from reaction, surface-initiated polymerization

## Abstract

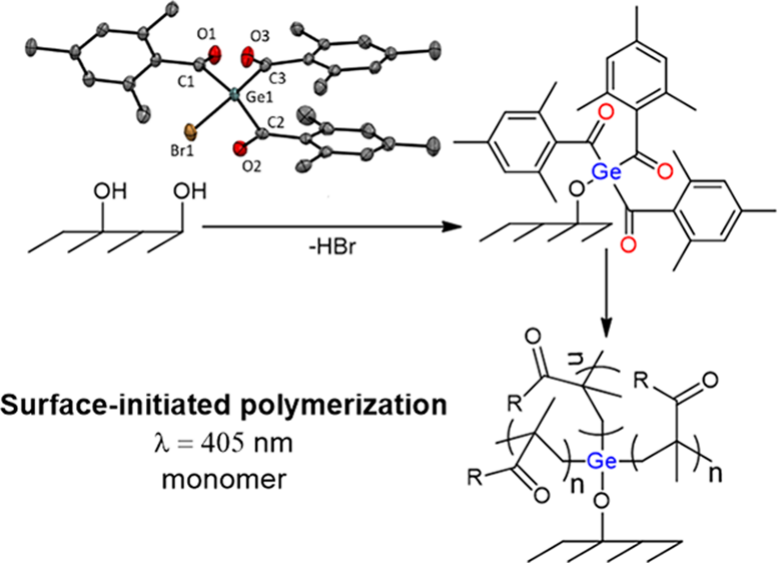

Since surface-initiated photopolymerization techniques have gained increasing interest within the last decades, the coupling of photoinitiators to surfaces and particles has become an important research topic in material and surface sciences. In terms of surface modification and functionalization, covalently coupled photoinitiators and subsequent photopolymerizations are employed to provide a huge variety of surface properties, such as wettability, stimulus responsive features, antifouling behavior, protein binding, friction control, drug delivery, and many more. For this purpose, numerous type I and type II photoinitiators or other photosensitive moieties have been attached to different substrates so far. In our studies, a convenient and straightforward synthetic protocol to prepare a novel germanium-based photoinitiator (bromo-tris(2,4,6-trimethylbenzoyl)germane) in good yields was developed. The immobilization of this photoinitiator at the surface of silicon wafers and quartz plates was evidenced by X-ray photoelectron spectroscopy (XPS). Employing visible-light-triggered surface-initiated polymerization of different functional monomers, including acrylamide, perfluorodecyl acrylate, and fluorescein-*o*-acrylate, surfaces with various features such as hydrophilic/hydrophobic and fluorescent properties were prepared. This was also achieved in a spatially resolved manner. The polymer layers were characterized by contact angle measurements, UV–vis/fluorescence spectroscopy, spectroscopic ellipsometry, and XPS. The thicknesses of the surface grafted polymer layers ranged between 10 and 126 nm.

## Introduction

In the last decades, surface modification techniques have become an incredibly interesting field of research in science as well as in the industrial sector. In most cases, surface modification aims to create a specific functionality that is able to interact with other media, materials, or components. Those functionalities include, for example, control of adhesion,^1^ wettability,^2,3^ adsorption properties,^4^ and anticorrosive^5^ or antifouling^6^ behavior of surfaces. Considering this wide range of applications, great efforts have been made in the biomedical field. For example, surface properties of nanodrug delivery systems play a huge role with respect to their effectiveness. In this context, tailoring the surface properties can lead to prolonged circulation times, increased loading capacity, enhanced accuracy of targeting infectious sites, enhanced cellular penetration, and many more.^7^ To give another example, interactions between human cells and implant materials are strongly dependent on surface parameters and are therefore heavily considered in implant design.^8^ Successful surface modification can be done in various ways, reaching from etching with highly reactive species, e.g., ozone or oxygen plasma, over silanization and vapor deposition methods over (spatially resolved) polymer coatings for high end applications. Overviews of different surface modification techniques applicable for various materials and even enzymes are given in refs (9−13). Among all these surface modification methods, coatings based on polymers are used in different applications and industries, as they provide a huge number of diverse functionalities for the underlying substrates.^14^

**Figure 1:**
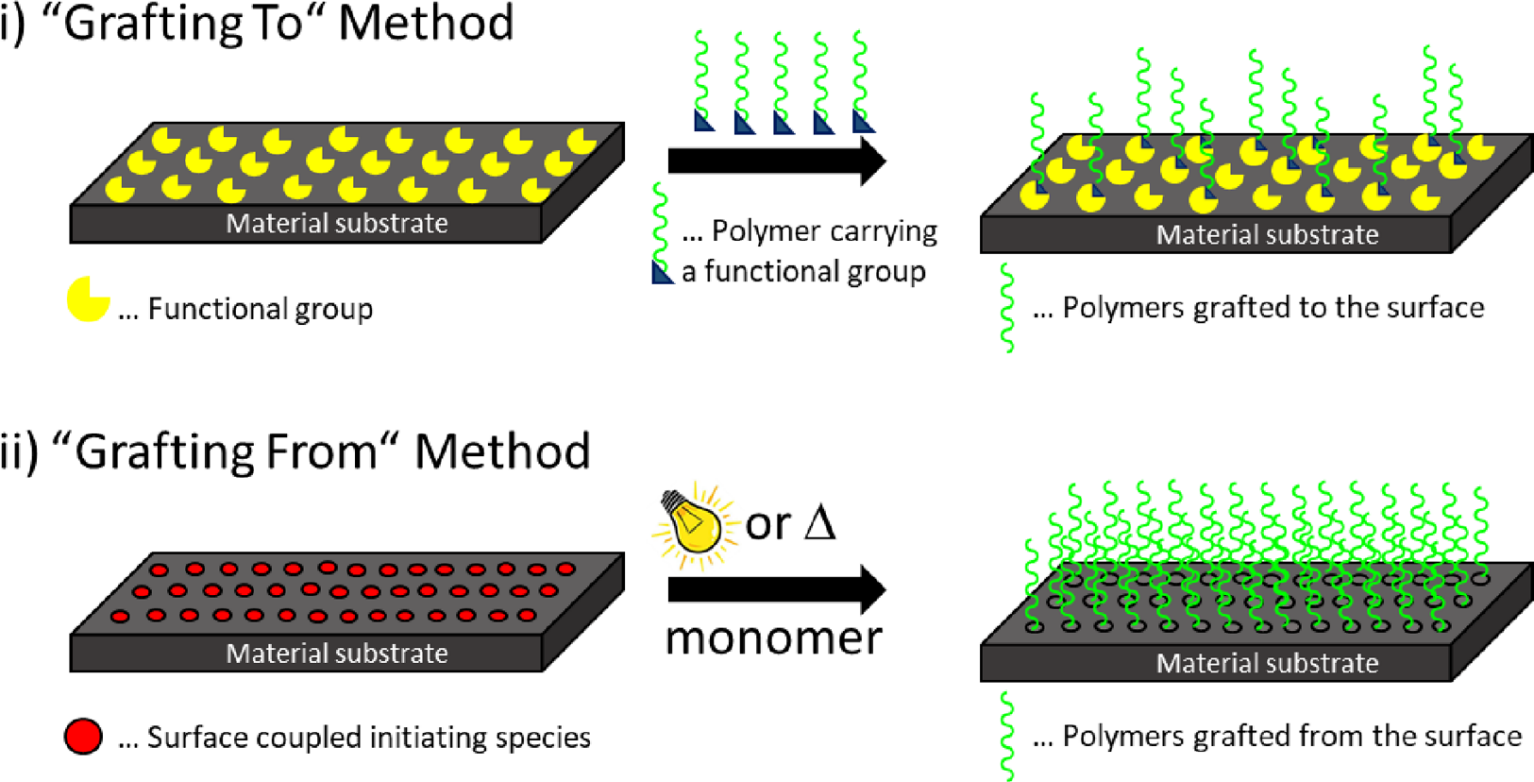
Schematic illustration of surface modification using “grafting to” and “grafting from” techniques.

In general, surface modification with polymeric layers can be done by both physical and chemical modifications. Physical modification suffers from severe limitations including low thermal stability, the inability to withstand high shear forces, and the fact that the applied layers can be easily removed by appropriate solvents. Chemical modification, on the other hand, is more desirable because it leads to more stable systems due to covalent linkage. This technique is mainly carried out via “grafting from” or “grafting to″ reactions. A schematic representation of both processes is depicted in Figure 1. The “grafting to” approach, where preformed polymeric macromolecules are attached to the surface, is achieved by exploiting a chemical reaction between two functional groups, one of which is already tethered to the surface and the other one is part of the macromolecule. Furthermore, using surface-coupled photosensitive species, such as phenyl azides, light-triggered (cyclo) addition or insertion reactions with other molecules can also be employed for the “grafting to” approach. The “grafting from” technique, which, in comparison to the “grafting to” counterpart, leads to higher grafting densities and thicknesses, directly initiates and therefore starts a polymerization from the surface. This is based on the formation of highly reactive species such as radicals or ions derived from surface tethered initiators upon heat exposure or irradiation.^10,15^ Therefore, much progress has been made to covalently couple (photo)initiating species to different surfaces. An overview of these reactions, including numerous initiating species and coupling strategies, is given in one of our previous contributions.^10^

Whereas the “grafting to” method requires the usage of a preformed polymer, numerous surface-initiated polymerization reactions are available for the “grafting from” method, including, for example, simple radical polymerization, surface-initiated photoiniferter-mediated polymerization (SI-PIMP),^16,17^ surface-initiated atom-transfer radical polymerization (SI-ATRP),^18^ as well as surface-initiated photoinduced electron/energy transfer reversible addition–fragmentation chain transfer polymerization (SI-PET-RAFT).^19−21^

Among numerous polymerization techniques, especially light-induced processes have received much interest in the last couple of years because they exhibit pronounced advantages compared to thermal initiation. The high reaction rate, low process cost, and energy efficiency make light-triggered polymerization processes also desirable for industrial applications. However, first and foremost, the possibility to spatially and temporally control the polymerization simply by switching on and off the light is the most outstanding feature of this method.^10,15,22^

**Scheme 1:**
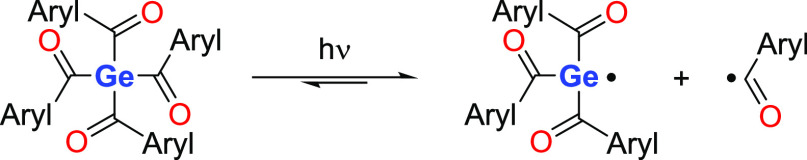
Photolytic Cleavage of Acylgermanes following the Norrish Type 1 Pathway

Novel germanium-based photoinitiators (acylgermanes) have shown to act as promising and efficient scaffolds for radical photopolymerization reactions. Like other type I photoinitiators, those compounds cleave via a Norrish I pathway (cleavage of Ge–C(O) bond), resulting in highly reactive germanium centered radicals (germyl radicals), which readily add to monomeric double bonds and therefore start a polymerization reaction.^23−25^ A schematic representation of the reversible radical generation is provided in Scheme 1.

**Scheme 2:**
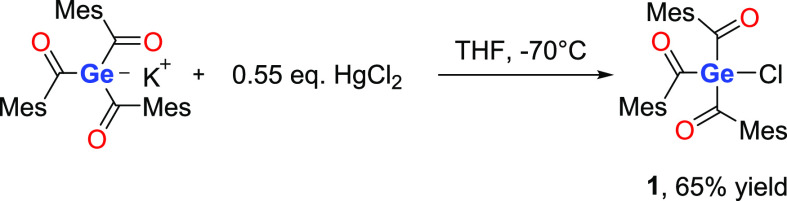
Synthesis Protocol for the Formation of **1**

Compared to well-established and widely used acylphosphine oxides, acylgermanes exhibit pronounced advantages. These include nontoxicity, high reactivity toward monomers, and the fact that the absorption spectra extend up to 480 nm. Particularly, the low toxicity in combination with visible light excitation opens up new applications in respect to biomedicine and long wavelength curing.^26^ For these reasons, a lot of research in terms of acylgermanium synthesis has been performed. Recently, it was found that chloro-tris(2,4,6-trimethylbenzoyl)germane is accessible, which represents an ideal precursor for surface immobilization (Scheme 2). However, in this approach, mercury(II) chloride was used in slight excess to synthesize this chlorine substituted trisacylgermane.^27^

Because mercury(II) chloride is considered to be acutely as well as cumulatively poisonous, a different synthetic approach toward halogenated trisacylgermanes was highly desirable.

Herein, we report the synthesis of a novel bromo-trisacylgermane **2**, which was accessed by a more moderate reaction approach and characterized by NMR spectroscopy, single-crystal X-ray crystallography, and UV–vis spectroscopy. Furthermore, this compound was immobilized onto silicon and quartz surfaces via a convenient and straightforward procedure. Moreover, we proved the “grafting from” capability of this covalently tethered photoinitiator through surface initiated photopolymerization reactions of different functional monomers, thereby preparing hydrophobic, hydrophilic, and fluorescent polymer layers. The choice of monomers was motivated by the fact that hydrophobic surfaces are widely discussed in literature for, among many others, antiadhesive and antifouling applications. Also, hydrophilic surfaces are important for different applications such as protein immobilization, whereas fluorescent tags are useful to introduce visibility-on-demand-properties to coated surfaces. In addition to full surface polymerization, spatially resolved patterns of the different polymer layers were prepared as well.

## Experimental Part

### Materials

For synthesis, all experiments were performed under a nitrogen atmosphere using standard Schlenk techniques. Solvents were dried using a column solvent purification system.^28^ Me_3_SiCl (≥99%), GeCl_4_ (>99.99%), KO*t*Bu (>98%), ClC(O)Mes, 1,2-dibromoethane (≥99%), and benzene-*d*_6_ (99.5 at. %, D) were all purchased from Sigma Aldrich and used without any further purification. For the measurements of air-sensitive samples, benzene-*d*_6_ was additionally dried by 8 h reflux above a sodium/potassium alloy. Tetrakis(trimethylsilyl)germane^29^ and potassium-tris(2,4,6-trimethylbenzoyl)germanide·0.5 DME^30^ were prepared according to published procedures.

*N*,*N*-Diisopropylethylamine (99%, DIPEA), anhydrous toluene (99.8%), fluorescein-*o*-acrylate (97%, FlAcr), 1-pyrenemethyl methacrylate (99%, PyrMAcr), acrylamide (99%, AAm), and 1*H*,1*H*,2*H*,2*H*-perfluorodecyl acrylate (97%, PFAcr) were all purchased from Sigma Aldrich and used without any further purification. Anhydrous tetrahydrofuran (99.9%, THF) was also obtained from Sigma Aldrich and distilled before use in polymerization experiments. Hexafluoroisopropanol (99%, HFIP) was purchased from ABCR and used as received.

Optically polished quartz plates were obtained from Korth Kristalle GmbH (Altenholz, Germany), and silicon wafers with one polished side were kindly provided by Infineon Technologies Austria AG (Villach, Austria).

### Synthesis of Bromo-tris(2,4,6-trimethylbenzoyl)germane (**2**)

To a solution of 3.99 mL dibromoethane (45.88 mmol, 5.50 equiv) in 20 mL of THF, 5.00 g potassium-tris(2,4,6-trimethylbenzoyl)germanide·0.5 DME (8.34 mmol, 1.00 equiv) in 70 mL of THF was added at −70 °C. The mixture was allowed to warm up to room temperature. Subsequently, the solvent was removed in a vacuum, and the product was resuspended in toluene and filtrated using a syringe filter. Then, the solvent was again removed, and the crude product was dissolved in hot *n*-heptane. The insoluble precipitate was filtered off and fully characterized as side product **2a**. Compound **2** was crystallized from the filtrate at −30 °C and isolated by filtration.

**2**: **yield**: 3.66 g (6.16 mmol; 73.85%) of analytically pure **2** as yellow crystalline solid. **mp**: 100–105 °C. **Anal. Calc** (%) for C_30_H_33_BrGeO_3_: C, 60.65; 5.60 H, found: C, 60,72; H, 5.62. **^13^C NMR data** (benzene-*d*_6_, TMS, ppm): 227.40 (*C*=O), 140.18, 140.14, 133.58, 129.21 (Mes-*C*), 21.08, 19.63 (aryl-*C*H_3_). **^1^H NMR data** (benzene-*d*_6_, TMS, ppm): 6.46 (s, 6H, Mes-*H*), 2.23 (s, 18H, Mes-C*H*_3_), 1.96 (s, 9H, Mes-C*H*_3_). **UV–vis**: λ [nm] (ε [L mol^–1^ cm^–1^]) = 402 (1455), 381 (1822). **IR** (neat): ν(C=O) = 1655, 1638, 1606.

**2a**: **yield**: 151 mg (223 μmol; 2.67%) of analytically pure **2a** as yellow crystalline solid. **mp**: 132–142 °C. **Anal. Calc** (%) for C_40_H_44_GeO_5_: C, 70.92; 6.55 H, found: C, 70.69; H, 6.58. **^13^C NMR data** (benzene-*d*_6_, TMS, ppm): 228.26 (Ge-*C*=O), 173.26 (Ge-O(*C*=O)Mes), 141.35, 139.93, 134.19, 129.29 (Mes-*C* of Mes(*CO*)Ge), 139.31, 137.09, 130.50, 129.05 (Mes-*C* of Mes(*CO*)OGe), 21.06, 19.43 (Aryl-*C*H_3_ of Mes(*CO*)Ge), 20.99, 20.49 (Aryl-*C*H_3_ of Mes(*CO*)OGe). **^1^H NMR data** (benzene-*d*_6_, TMS, ppm): 6.56 (s, 2H, Mes-*H*), 6.48 (s, 6H, Mes-*H*), 2.35 (s, 6H, Mes-C*H*_3_), 2.31 (s, 18H, Mes-C*H*_3_), 2.00 (s, 9H, Mes-C*H*_3_), 1.95 (s, 3H, Mes-C*H*_3_). **UV–vis**: λ [nm] (ε [L mol^–1^ cm^–1^]) = 400 (1059), 377 (1598), 353 (998), 284 (20820). **IR** (neat): ν(C=O) = 1671, 1662, 1642, 1605.

### Surface Functionalization of Silicon Substrates and Quartz Plates with Bromo-tris(2,4,6-trimethylbenzoyl)germane (**2**)

Silicon wafers and quartz plates were immersed into acetone and isopropanol, respectively, and ultrasonicated in each solvent for 10 min. After washing, both types of substrates were pretreated by exposure to oxygen plasma using a plasma etching system from Oxford Instruments (Abingdon, United Kingdom). The treatment was carried out for 2 min with 100 W, 40 mTorr oxygen pressure, and an oxygen flow of 50 cm^3^ min^–1^ For the quartz plates, this treatment was repeated to activate both the top and bottom side of the plates. Silicon wafers were treated on the polished, reflecting side only.

The activated substrates were then immediately immersed in a filtered THF solution of compound **2** (45.45 mmoL/L). Additionally, an excess of Hünig’s base (diisopropylethylamine, 2.4 equiv) was added to scavenge hydrogen bromide to avoid unwanted side reactions. The reaction was carried out under exclusion of light and under an inert gas atmosphere at 60 °C for 24 h. After the coupling process, the samples were washed with anhydrous THF and toluene.

### Surface-Initiated Photopolymerization

The functionalized quartz plates were placed in a 50 mL round flask (made from borosilicate glass) containing a solution of 1-pyrenylmethyl methacrylate or fluorescein-*o*-acrylate (PyrMAcr or FlAcr, both 353 mmoL/L) in distilled and degassed THF. Subsequently, the samples were illuminated with λ = 405 nm through the flask under an inert gas atmosphere for 10 min. A LED light source (Opsytec Dr. Gröbel, Germany) with 100 W was used, and the distance between the light source and sample was 5.5 cm (light intensity of approx. 10.3 W/cm^2^ at the sample surface). After illumination, the samples were washed thoroughly three times (2 min each) with THF under ultrasonication.

Moreover, films of poly(1*H*,1*H*,2*H*,2*H*-perfluordecyl-acrylate) (p(PFAcr)) and poly(acrylamide) (p(AAm)) were grafted from silicon wafers bearing the initiating group at the surface. Therefore, the functionalized silicon wafers were placed in an illumination chamber equipped with a gas inlet and outlet and a quartz window for illumination from the top. Then, the respective monomer was drop coated onto the functionalized wafer. Whereas PFAcr was used without dilution, AAm was diluted with EtOH (50 mg of AAm in 200 μL of EtOH). After drop coating, the chamber was closed and flushed with nitrogen for several minutes. Surface initiated photopolymerization was then carried out upon irradiation with λ = 405 nm and 100 W at a distance of 5 cm for 1 min (PFAcr, 1.032 W/cm^2^) or 1.5 min (AAm, 1.548 W/cm^2^), respectively. Finally, the samples were ultrasonicated (a) for 2 min in HFIP, DCM, and acetone for p(PFAcr) or (b) for 10 min in EtOH for p(AAm), respectively, to remove the nonreacted monomer.

### Spatially Resolved Photopolymerization

Spatially resolved polymer films of p(PFAcr) were generated on **2**-modified silicon wafers. In a first step, the immobilized germanium-based photoinitiator on the surface was locally deactivated by UV exposure. Therefore, the silicon wafers were covered by a photomask (see Scheme 5) and irradiated under ambient conditions with λ = 405 nm and 100 W at a distance of 5 cm for 20 s, employing the same lamp as described before. After 10 min, the sample with the photomask on top was irradiated again for 20 s with the same parameters. This cycle was then repeated for one more time so that the final illumination time was 60 s (light intensity at the sample surface 1.032 mW/cm^2^). In our laboratories, this procedure proved to be the most efficient one to locally deactivate the photoinitiating species. Because no monomers or other species were placed between the sample and the mask during the deactivation step, we assume that oxygen from the air is needed to react with the surface radicals produced by the photoinitiator upon illumination. By interrupting the illumination for 10 min, oxygen is allowed to diffuse between the sample surface and the photomask.

After this spatially resolved deactivation step, surface initiated photopolymerization was carried out in the illumination chamber as described above. It has to be mentioned here that the monomer was drop coated all over the wafer. However, photopolymerization was only possible at the regions that were shielded during the deactivation step and thus still contain active photoinitiator groups. Finally, the samples were ultrasonicated for 2 min in HFIP three times and once in DCM and acetone, respectively.

In addition, polymer films with combined structures of p(PFAcr) and p(AAm) were also prepared on silicon wafers. For this purpose, functionalized wafers were placed in the illumination chamber, drop coated with AAm (dissolved in EtOH; 50 mg in 200 μL), and covered by different photomasks. The surface initiated photopolymerization was carried out under inert gas applying a light intensity on the surface of approximately 1.548 W/cm^2^ as described above. Subsequently, the partially polymerized wafers were ultrasonicated in EtOH for 10 min and dried at 60 °C. In a second step, the structured wafers were again placed in the chamber and drop coated with PFAcr. Another surface initiated photopolymerization step was carried out under the same conditions as described for the polymerization of PFAcr (1.032 W/cm^2^). Finally, the samples were ultrasonicated in HFIP, DCM, and acetone for 2 min and subsequently dried.

### Characterization Methods

#### NMR Spectroscopy

^1^H and ^13^C NMR spectra were recorded on a Varian INOVA 300, a 200 MHz Bruker AVANCE DPX, or a Bruker Avance 300 MHz spectrometer in benzene-*d*_6_ solution and referenced to tetramethylsilane (TMS) using the internal ^2^H-lock signal of the solvent.

#### Melting Point Measurement

Melting points were determined using a Stuart SMP50 apparatus and are uncorrected.

#### Elemental Analysis

Elemental analyses were carried out on a Hanau Vario Elementar EL apparatus (Hanau, Germany).

#### FTIR Spectroscopy

For analysis of pure **2** as a solid sample, infrared spectra were recorded on a Bruker Alpha-P Diamond ATR Spectrometer.

Further infrared spectroscopy was conducted using an FTIR spectrometer from Bruker Optics, Germany (model Vertex 70). The spectra were recorded from solid samples using an ATR unit with a total number of 16 scans (resolution 1 cm^–1^).

#### X-ray Crystallography

X-ray crystallography was performed on a Bruker APEX II diffractometer with the use of an Incoatec microfocus sealed tube of Mo Kα radiation (λ = 0.71073 Å) and a CCD area detector.

#### UV–Vis Spectroscopy

UV–vis absorption spectra were recorded on a Perkin Elmer Lambda 5 spectrometer. The fluorescent polymer films that were grafted from the quartz plate surfaces were characterized by means of their specific absorption bands employing a Cary 50 UV–vis spectrometer (Varian, Australia), which was equipped with a xenon-flashlight lamp. For measuring in transmission, the samples were placed on a proper sample holder. Because the polymerization process is expected not to significantly change the absorption properties of the chromophore moiety, the absorption spectra of the grafted polymer films were compared to those of the corresponding monomers in the solution, which were measured in THF at different concentrations using 1 cm quartz cuvettes. From these data, molar decadic absorption coefficients of the monomers were calculated according to the Lambert–Beer equation.

#### Fluorescence Spectroscopy

Fluorescence emission spectra of the polymer coated quartz plates were investigated using a Cary Eclipse spectrometer (Varian, Australia). The samples were excited by UV irradiation with a scan rate of 600 nm/min. For this purpose, the quartz plate with poly(fluorescein-*o*-acrylate) was excited at 405 nm, and the plate with poly(pyrenylmethylmethacrylate) was excited at 347 nm. In general, the strength of the fluorescent signals of the coated quartz plates is strongly dependent on the geometric position of the plates in the sample holder. Therefore, these measurements only provided qualitative information. The recorded fluorescence spectra were compared to the fluorescence spectra of the corresponding monomers in THF solutions.

#### X-ray Photoelectron Spectroscopy (XPS)

The chemical composition of the sample surfaces was analyzed by XPS at room temperature using a Nexsa G2 Surface Analysis System from ThermoFisher Scientific with monochromatic Al Kα radiation. Survey scans were carried out at a pass energy of 200 eV and an energy resolution of 1.0 eV, whereas high-resolution spectra were recorded at a pass energy of 50 eV and a resolution of 0.1 eV. The C1s line was used to calibrate the binding energy scale for the measurements, assuming a binding energy of 284.8 eV for C–C bonds. Hydrogen was omitted in the calculation of the surface composition. For each sample, at least two measurements were performed.

#### Contact Angle Measurements

Moreover, the contact angles of the sample surfaces with water (WCA) were recorded at room temperature with a drop shape analyzer (DSA 100, Krüss GmbH, Germany). Ultrapure water droplets with a volume of 2 μL were deposited on the sample surfaces and evaluated using the software “Drop Shape Analysis” from the same supplier. The WCA was averaged over two to three measurements on each sample.

#### Spectroscopic Ellipsometry

Ellipsometric measurements were performed to determine the thickness of the deposited polymeric layers. An ex situ variable angle spectroscopy ellipsometer (model M-2000 from J.A. Woollam Corp., Lincoln, USA) was used for these measurements.

## Results and Discussion

**Scheme 3:**
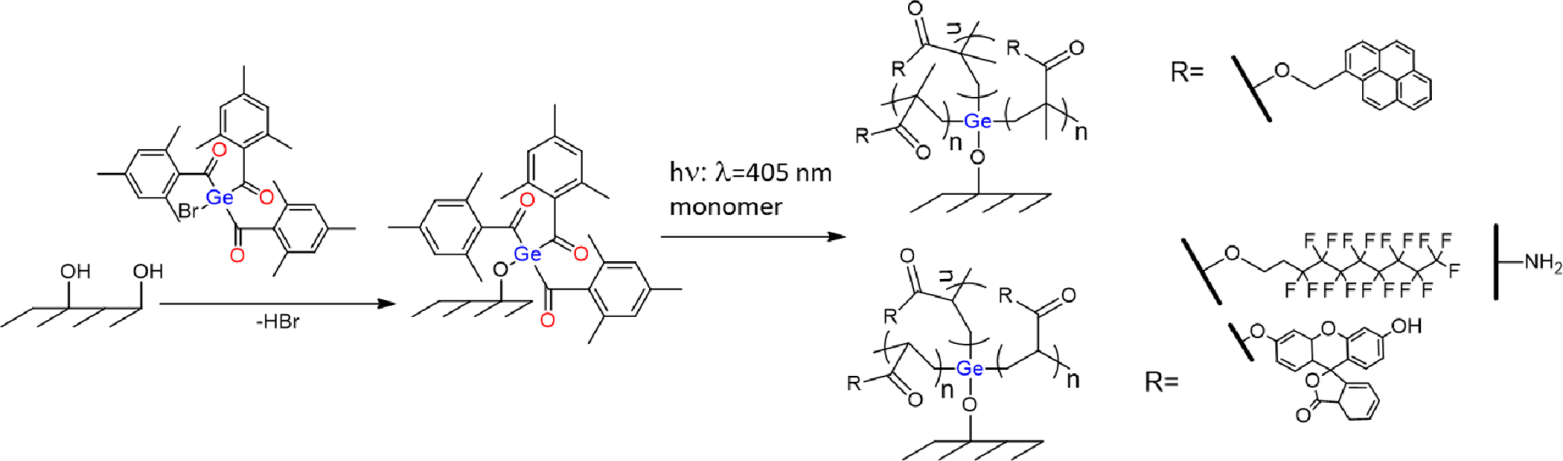
Schematic Presentation of the Covalent Coupling of **2** onto Oxygen Plasma Activated Surfaces with Subsequent “Grafting From” Photopolymerization of Functional Monomers

Herein, we report a facile and convenient method for a photoinduced “grafting from” polymerization on inorganic surfaces such as silicon wafers and quartz plates, which was achieved via the immobilization of a novel germanium-based photoinitiator **2**. As shown in Scheme 3, those surface tethered species were exploited to initiate a radical polymerization reaction of different functional monomers upon irradiation with visible light (405 nm).

**Scheme 4:**
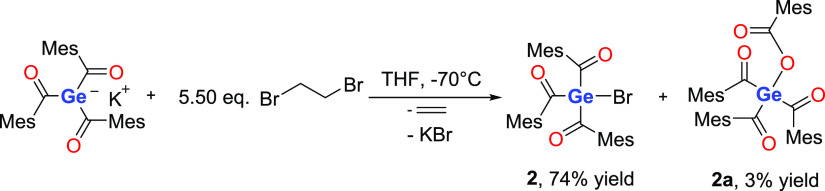
Reaction of Triacylgermenolate with 1,2-Dibromoethane

The starting point for this investigation was the straightforward synthesis of the bromo-tris(2,4,6-trimethylbenzoyl)germane **2** as outlined in Scheme 4. During the reaction, a metal-halogen exchange reaction takes place forming the target compound, ethylene, potassium bromide, and minor traces (<5%) of **2a** as side products. On the basis of the reaction depicted in Scheme 4, only 1.0 equiv of 1,2-dibromoethane would be sufficient to ensure a complete conversion to compound **2**. However, we observed a significant suppression of the formation of side products by the use of 1,2-dibromoethane in excess. Fine-tuning of the reaction conditions and temperature increased the isolable yield to 74%.

The purity of compound **2** was confirmed by NMR spectroscopy (detailed assignments are provided in the Materials and Characterization Methods sections; NMR spectra are provided in the Supporting Information, see Figures S1–S4). For acylgermanes, the ^13^C NMR shift for the carbonyl moiety is a characteristic feature. For compound **2**, this ^13^C shift was found at δ = 227.40 ppm. This value corresponds well to the ^13^C signals of carbonyl moieties, which are directly linked to a germanium atom as can be seen in ref (27). Compared to NMR shifts of related acylgermanes, on the other hand, a high field shift is noticed, being related to the attached halogen unit (Table 1; **1,**(27)**3,**(25)**4**,^30^ and **5**(31)).

**Table 1:**
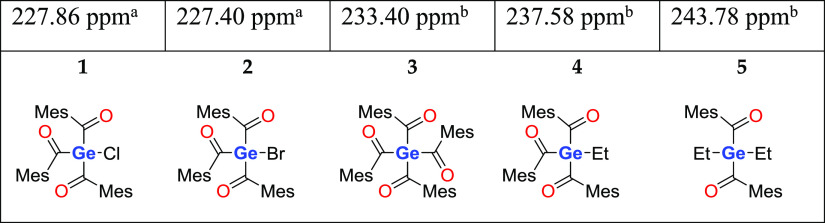
Comparison of ^13^C NMR Carbonyl Shifts of Acylgermanes **1**–**5**

a Measured in C_6_D_6_.

b Measured in CDCl_3_.

**Figure 2:**
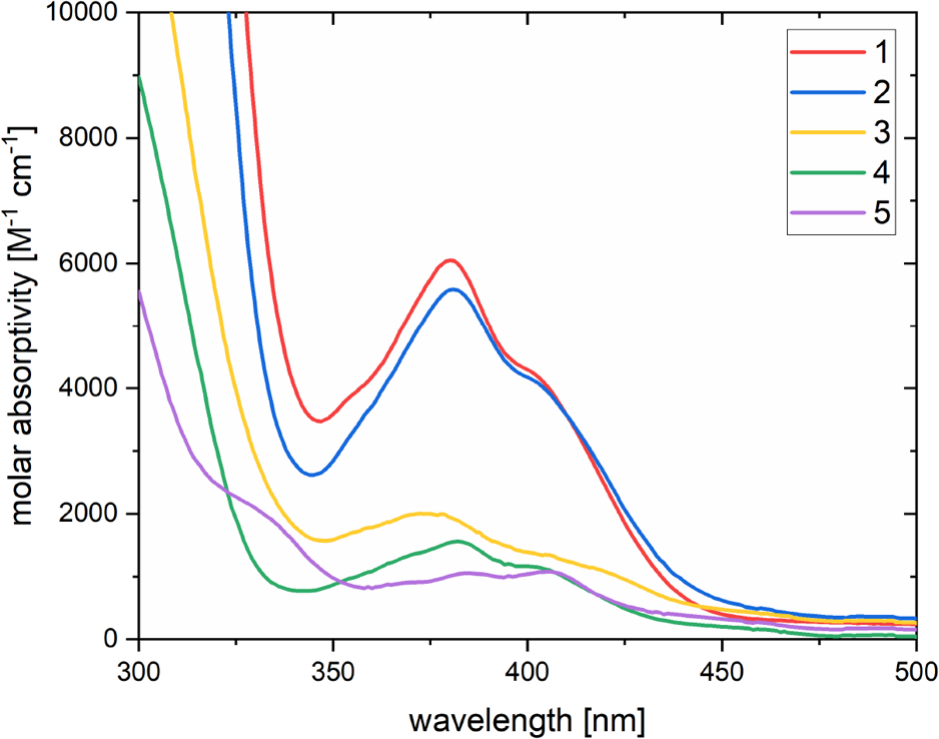
Comparison of UV–vis absorption spectra of compounds **1–5** (*c* = 1 × 10^–4^ M).

By comparing the UV–vis absorption spectra of bromo-tris(2,4,6-trimethylbenzoyl)germane **2** with those of other acylgermane derivatives, it was noticed that, in accordance with compound **1**, which also carries a halogen atom, a significantly increased absorption can be observed (Figure 2). We assume that the attached halogen atoms are responsible for this high intensity due to an increased oscillation strength of the n-pi* transition. However, this effect is currently under investigation and will therefore not be further discussed within this contribution.

**Figure 3:**
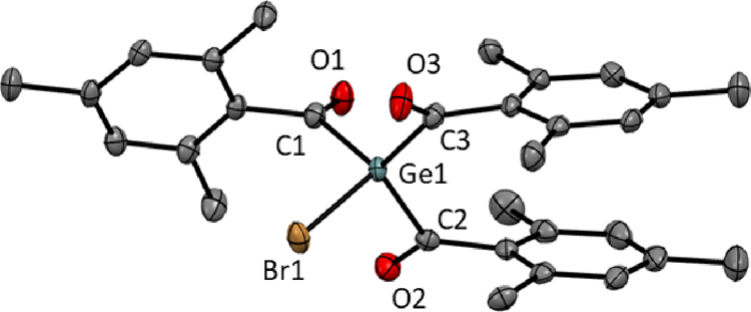
ORTEP representation of compound **2**. Thermal ellipsoids are depicted at the 50% probability level. Hydrogen atoms are omitted for clarity. Selected bond lengths (Å) and bond angles (°) with estimated standard deviations: Ge(1)-Br(1) 2.3164(3), Ge(1)-C(1) 2.037(2), Ge(1)-C(2) 2.044 (2), Ge(1)-C(3) 2.049(2), C(1)-O(1) 1.204(2), C(1)-O(2) 1.208(2), C(1)-O(3) 1.205(3), C(1)-Ge(1)-C(2) 100.36(8), C(1)-Ge(1)-C(3) 121.26(8), C(2)-Ge(1)-C(3) 121.07(8), C(1)-Ge(1)-Br(1) 104.49(6).

Additionally, single crystals suitable for X-ray analysis were obtained by cooling the concentrated solution of **2** in THF to −30 °C. The corresponding representation is given in Figure 3. It was found that compound **2** crystallizes in the monoclinic space group *P*2_1_/*c* and contains four molecules per unit cell. In comparison to the chloro-substituted derivative **1**, similar bond lengths for the Ge–C and C=O bonds were found.^32^ Moreover, the central germanium atom is also pyramidal.

### Immobilization of the Photoinitiator and Surface-Initiated Polymerization Reactions

The immobilization of compound **2** onto silicon substrates was accomplished in two steps. The first step comprised surface activation with oxygen plasma, resulting in hydroxy terminated silicon and quartz surfaces, respectively.^33−35^ Whereas the silicon wafers were only activated and modified on the polished surface (top), the quartz plates were activated on the top and bottom sides to couple the photoinitiator to both sides. Immobilization of initiating species was carried out by immersing the previously activated substrates in a THF solution of compound **2** and DIPEA at 60 °C for 24 h. We propose that this reaction follows a nucleophilic substitution pathway according to Scheme 3. This is based on literature reports on the reactions of bromo-substituted germanes with alcohols in the presence of bases to yield the respective hydroxy and alkoxy derivatives.^36^ In addition, the formation of a tertiary ammonium salt (*N*-ethyl-*N*-isopropylpropan-2-ammonium bromide) further supports the suggested reaction pathway. The ammonium salt was obtained as a side product derived from the reaction between DIPEA and hydrogen bromide, which in turn was generated as a consequence of the reaction between the surface hydroxyl groups and compound **2**. This solid white powder was characterized with FTIR spectroscopy, revealing a group of sharp and strong absorption bands at 2684, 2664, and 2618 cm^–1^ due to hydrohalide N-H^+^ stretching vibrations. The corresponding FTIR spectra of this compound is included in the Supporting Information (Figure S8).^37^

**Figure 4:**
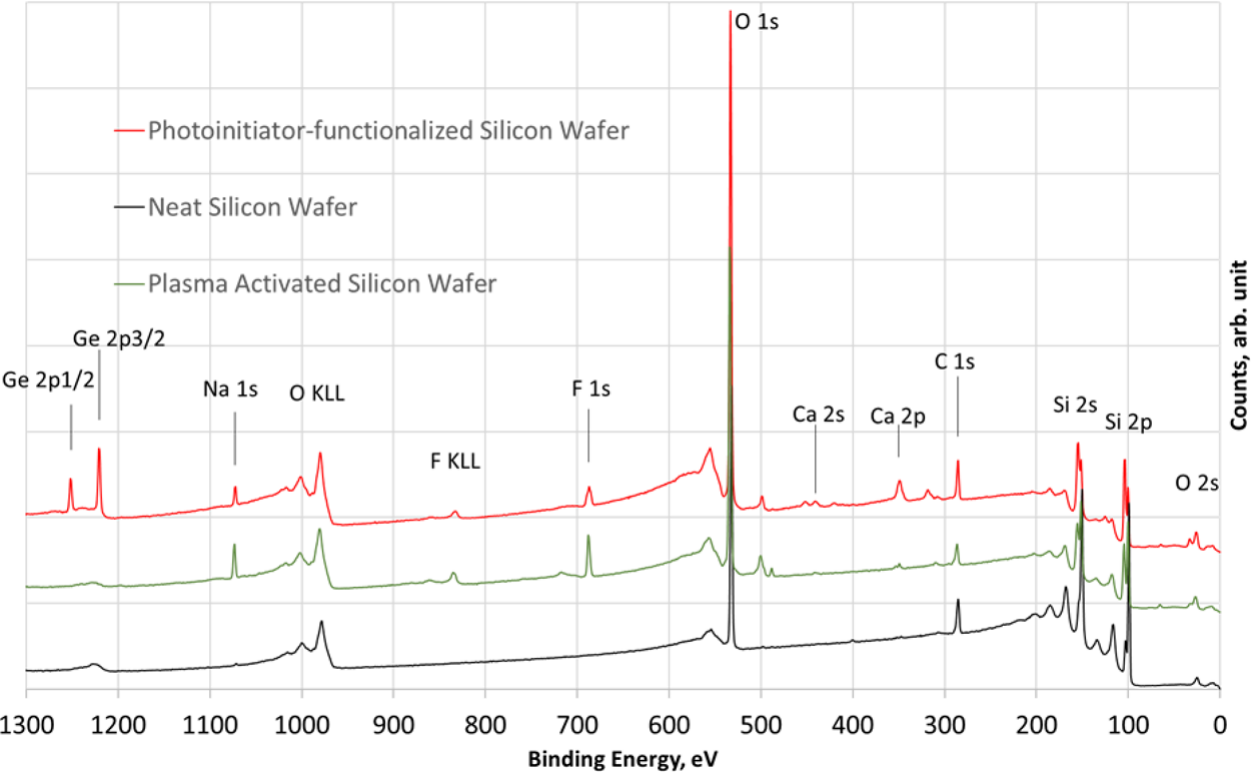
XPS survey spectra including peak assignments of a neat Si wafer (bottom, black curve) and oxygen plasma activated and photoinitiator-functionalized Si wafers (green and top red curve).

The immobilization of the photoinitiator was proven by XPS analyses of the functionalized silicon wafers, and the signal peaks were identified with reference data.^38^ As can be seen from the survey spectra in Figure 4, the presence of Ge (1220 and 1251 eV) and the absence of Br (69 eV) were evidenced, strongly suggesting the successful covalent linkage of the initiating species following the above described pathway. Moreover, the elemental composition of silicon wafers before and after surface functionalization is compared in Table 2. Surprisingly, the carbon content of the functionalized substrate was much too low with regard to the molecular architecture of compound **2**. This was explained by the fact that the samples were inevitably illuminated with white light in the XPS instrument during the adjustment of the height between the electron gun and the detector. A partial decomposition of the visible light sensitive moiety of the photoinitiator on the surface is expected to start during the alignment procedure. Furthermore, some traces of impurities were observed at the surface, including Na, F, and Ca atoms. However, these elements were also detected on neat silicon wafers after activation with oxygen plasma without further surface functionalization (see Figure 4, green curve). It is also worth mentioning that a significant amount of carbon was found on neat silicon wafers too. This was assigned to absorbed carbonaceous species including carbon dioxide, carbon monoxide, etc., as already documented in a previous work.^1^

**Table 2:** Relative Atomic Surface Composition of a Neat and a Functionalized Si Wafer (Data Obtained from High-Resolution XPS Spectra)

	surface composition [at. %]						
sample	C	Ge	F	O	Si	N	Na
neat Si wafer	14.7 ± 0.6			32.3 ± 0.34	51.6 ± 0.0	0.9 ± 0.1	
Si wafer functionalized with photoinitiator moieties	10.4 ± 0.5	2.5 ± 0.1	2.9 ± 0.1	49.8 ± 0.3	33.4 ± 0.2		0.9 ± 0.0

To examine the efficiency of the tethered photoinitiator, surface initiated photopolymerization reactions with four different monomers were carried out upon visible light illumination. Therefore, the quartz plates functionalized with the initiator on both sides were immersed into solutions of FlAcr or PyrMAcr in THF and exposed to light (405 nm) for 10 min. p(PFAcr) and p(AAm), respectively, were grafted from photoactive silicon wafers by drop coating of those substrates with liquid monomers (or monomer solutions) and subsequent illumination with light (405 nm) for 1 and 1.5 min, respectively. Finally, all wafers were intensely rinsed with appropriate solvents for several times, dried, and then characterized with regard to their surface composition and individual surface properties.

**Scheme 5:**
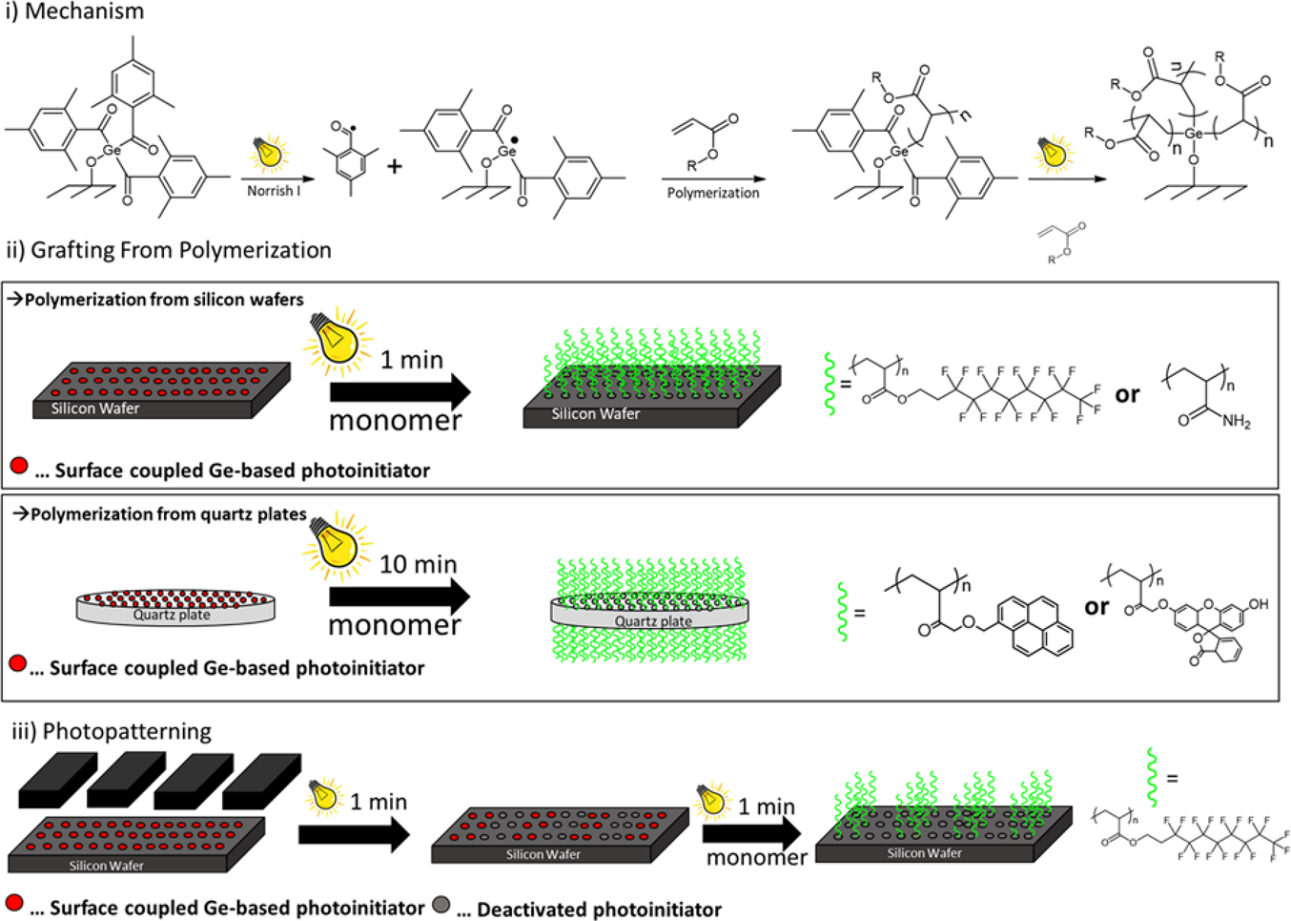
Schematic Representation of the Applied “Grafting From” Method (I) and the Prepared Polymer Layers, including Spatially Resolved Structures (II, III)

Upon visible light illumination, the initiating species at the surface undergo an α-cleavage process (Norrish type I reaction) once the molecule has reached its triplet state T_1_ (after intersystem crossing from the S_1_ state). As a consequence of each cleavage step, highly reactive germanium centered (germyl) radicals and far less reactive mesitoyl radicals are formed. The surface coupled germanium centered radical readily adds to (meth)acrylic double bonds and therefore starts the radical chain growth mechanism from the surface.^24,25^ Also, the less reactive mesitoyl radicals are expected to initiate polymerization reactions, which lead to the formation of noncoupled homopolymers.^15^ However, these noncoupled polymers can be easily removed by washing the polymer coated substrates. An overview of the mechanism and the prepared polymer layers on different substrates is provided in Scheme 5.

The grafted polymer layers p(FlAcr) and p(PyrAcr) on the quartz plates were characterized by XPS, proving the successful surface-initiated polymerization on both sides (top and bottom). As can be seen from Table 3, a comparison of the surface composition before and after modification of the quartz plates revealed significantly increased carbon contents, which were assigned to the coupled polymer layers of p(FlAcr) and p(PyrAcr), respectively. Furthermore, a decrease of O and Si was observed after polymerization, indicating the formation of a polymer layer on top of the quartz substrate. Also, the polymer-modified quartz plates showed a significantly increased amount of Ge in relation to Si when compared to silicon wafers functionalized with compound **2** (Ge/Si ratio of about 0.28 and 0.07, respectively; see Table 2). This can be explained as a result of the grafted polymer layer because of which less material of the quartz plate, consisting of Si and O, was measured, therefore enhancing the contribution of the Ge-rich interface to the overall atomic surface composition. However, because Si and Ge, which originate from the substrate functionalized with compound **2**, were still detected after polymerization, the polymer layers were expected to be thinner than the XPS penetration depth that is within the top 10 nm of the investigated surface.^39,40^ In addition, small amounts of Na, F, and Zn were also detected but are considered to be negligible here.

**Table 3:** Relative Atomic Surface Composition of a Neat Quartz Plate Compared to Quartz Plates after Surface Modification via Photoinduced “Grafting From” of Poly(fluorescein-*O*-acrylate) (p(FlAcr)) and Poly(pyrene methacrylate) (p(PyrMAcr))

	surface composition [at. %]						
sample	C	O	Si	Ge	Na	F	Zn
neat quartz plate	15.4 ± 3.0	60.6 ± 2.1	22.7 ± 1.3		1.3 ± 0.2		1.7^a^
quartz plate p(FlAcr)	32.2 ± 1.1	46.3 ± 0.9	15.6 ± 1.2	4.4 ± 1.1	1.0 ± 0.3	1.2^a^	
quartz plate p(PyrMAcr)	30.6 ± 1.8	47.0 ± 1.0	16.6 ± 0.8	5.5 ± 0.4	0.9^a^		

a Single value, detected on only one of four measurement spots on the sample.

**Figure 5:**
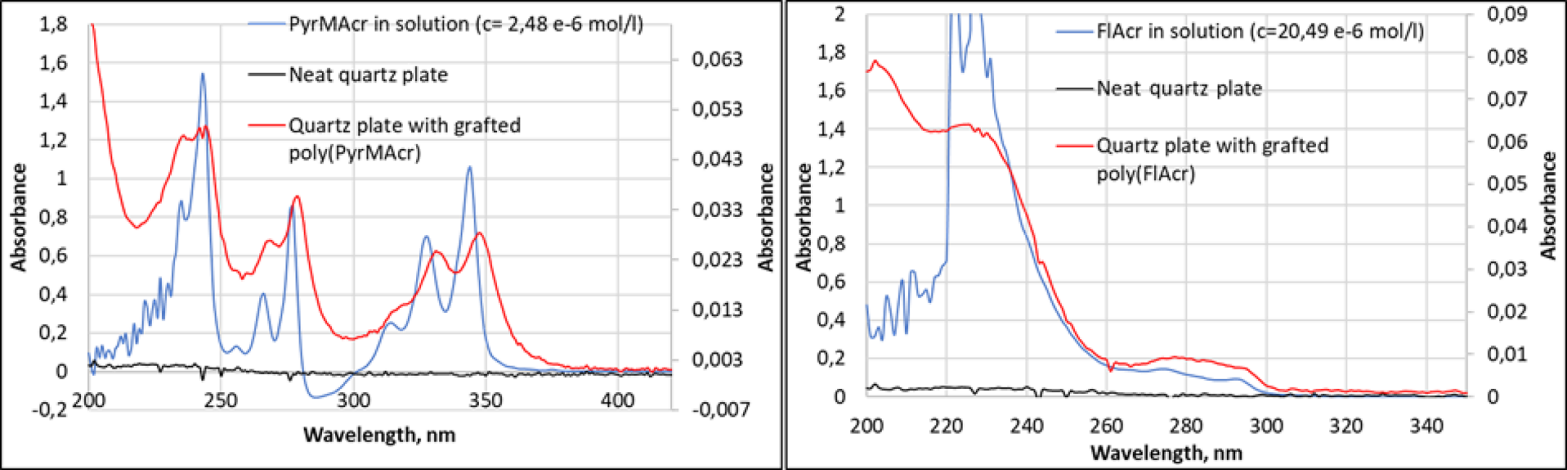
UV–vis absorption of neat and modified quartz plates compared with the corresponding monomers in solution (absorption of the modified quartz plates is plotted using the secondary *y* axis).

Besides XPS analysis, the presence of fluorescent polymer layers p(FlAcr) and p(PyrAcr) was proven by their specific UV–vis absorbance and fluorescence emission spectra. As can be seen from Figure 5, the UV–vis absorbance of the grafted polymer layers (see secondary *y* axis) shows a similar curve shape compared to the corresponding monomer in the solution. As the quartz plates were carefully rinsed before the measurements, this result evidenced the presence of immobilized polymers grafted from the quartz surfaces.

**Figure 6:**
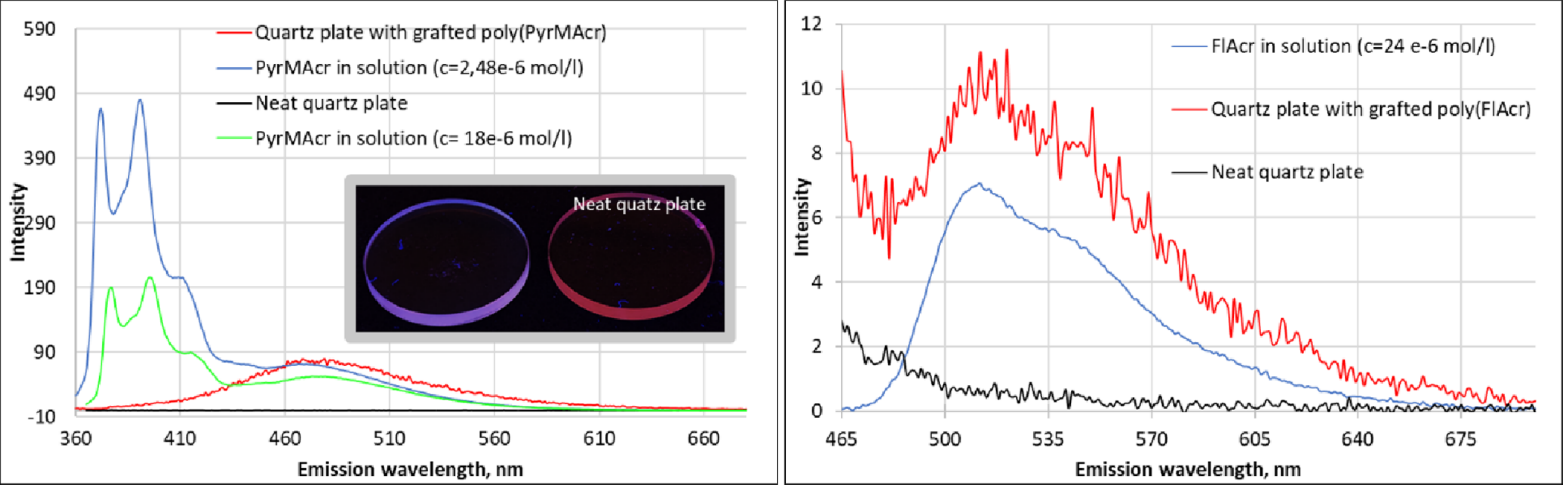
Fluorescence emission spectra of neat quartz plates compared to plates bearing grafted p(PyrMAcr) (right, 347 nm excitation wavelength) and p(FlAcr) (left, 450 nm excitation wavelength). The visible fluorescence upon excitation of grafted p(PyrMAcr) with 366 nm light is also shown (insert).

These results were supported by the fluorescence emission spectra, which are depicted in Figure 6. Notably, the quartz plate with grafted p(PyrMAcr) showed a strong and visible fluorescence upon excitation with UV light (λ = 366 nm), which is also shown in Figure 6. Visible fluorescence was not observed when exciting the quartz plate grafted with p(FlAcr) at different wavelengths. This is assigned to the fact that PyrMAcr shows a significantly higher molar decadic absorption coefficient of about 2.7 × 10^5^ l mol^–1^ cm^–1^ when compared to that of FlAcr (7.3 × 10^3^ l mol^–1^ cm^–1^, data from UV–vis measurements). However, when measuring the same plate employing a fluorescence spectrometer, a signal was observed that was in accordance with the signals of FlAcr in solution, thus also suggesting a successful grafting via photopolymerization. In contrast, fluorescence spectra of the quartz plate coated with p(PyrMAcr) revealed significant differences in respect to the spectra of the monomer in solution and the polymer layers (Figure 6). Especially the two sharp emission peaks in the region around 372 and 391 nm were only detected for the monomer solution but not observed for the polymer coated plate. In solution, these peaks decreased with increasing monomer concentration (see Figure 6, blue and green curves). This phenomenon was also observed by Förster and Kasper who performed fluorescence measurements of dissolved pyrene at different concentrations. With increasing concentrations, they observed an envelope of fluorescence, quenching violet fluorescence (peaks at 372 and 391 nm), which is accompanied by an appearance or increase of a broad blue fluorescence signal. This effect is attributed to singlet state excimer formation between an excited and unexcited pyrene molecule.^41^ This explains our results as the grafted polymer layers contain a high concentration of pyrene moieties, resulting in the red-shifted fluorescence signal (see Figure 6).

The grafting of PFAcr and AAm onto modified silicon wafer surfaces was proven by quantitative evaluation of the respective XPS survey spectra. As shown in Table 4, coupling of p(PFAcr) layers introduced a high amount of F (36.2 at. %), whereas the deposition of p(AAm) resulted in a significant amount of N (16.7 at. %). In addition, the C content increased from 10.4 at. % (functionalized wafer) to 26.5 at. % after polymerization of PFAcr and to 65.8 at. % after polymerization of AAm. In contrast, the Si, O, and Ge concentration decreased as the substrates were covered by the polymer layers. The fact that small amounts of Si and Ge were still detectable for the wafer covered with p(PFAcr) indicated a low thickness of the polymer layer. In contrast to this, for the p(AAm) sample, no substrate related elements were detectable.

**Table 4:** Overview of Atomic Surface Compositions Obtained from XPS Survey Spectra, and Water Contact Angles (WCAs) on a Neat Si Wafer, a Si Wafer Functionalized with Photoinitiator, and Si Wafers Bearing Photografted Layers of p(PFAcr) and p(AAm)

	surface composition [at. %]								
sample	C	Ge	F	O	Si	Cl	N	Na	WCA [°]
neat Si wafer	14.7 ± 0.6			32.3 ± 0.3	51.6 ± 0.0	1.3^a^	0.9 ± 0.1		45.1 ± 1.4
Si wafer functionalized with photoinitiator	10.4 ± 0.5	2.5 ± 0.1	2.9 ± 0.1	49.8 ± 0.3	33.4 ± 0.2			0.9 ± 0.0	58.6 ± 3.1
Si wafer with photografted p(PFAcr)	26.5 ± 2.3	0.7 ± 0.1	36.2 ± 5.9	22.4 ± 4.9	14.1 ± 3.2				124.5 ± 0.8
Si wafer with photografted p(AAm)	65.8 ± 0.6			17.5 ± 0.2			16.7 ± 0.7		11.8 ± 0.8

a Single value, detected on only one of 4 measurement spots on the sample.

**Figure 7:**
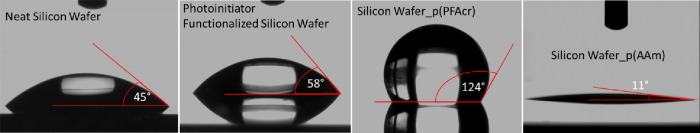
Water contact angles (WCAs) on different Si wafers. From left to right: neat silicon wafer, Si wafer functionalized with a photoinitiator, Si wafer with photografted p(PFAcr), and Si wafer with photografted p(AAm).

Supporting the results of XPS analysis, high and low water contact angles (WCAs, depicted in Figure 7) were obtained for p(PFAcr) and p(AAm) layers, respectively. The value of 124° significantly exceeds the initial WCA of the wafer functionalized with compound **2** (58°) and is typical for fluorinated polymer surfaces.^1,42,43^ In contrast, p(AAm) showed a low WCA (11°), which results from the polar amide moieties on the polymer surface interacting well with the polar test liquid (water).

**Figure 8:**
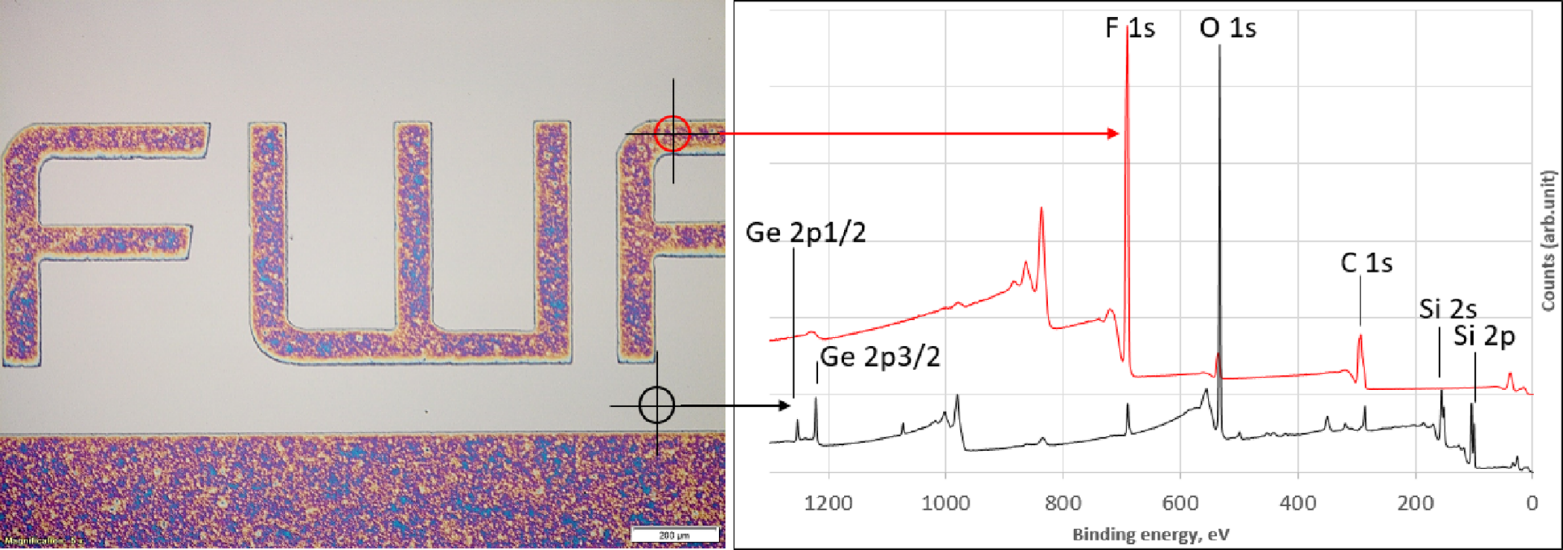
Microscope image of a patterned p(FAcr) film (colored areas) grafted on a Si wafer (gray areas are lacking a graft layer) and corresponding XPS survey spectra. The red curve is assigned to the area bearing a photografted polymer layer, and the black curve is assigned to areas lacking a graft layer.

The thicknesses of the p(PFacr) and p(AAm) layers were measured with spectroscopic ellipsometry. Those measurements revealed a thickness of 38 and 126 nm for p(AAm) and p(PFAcr), respectively. The significantly higher thickness of p(PFAcr) compared to that of p(AAm) is related to the fact that AAm was polymerized in the diluted phase (see Experimental Part), thus limiting the polymerization degree and, in further consequence, the layer thickness.^44^ However, the obtained thicknesses correspond well with already reported layer thicknesses (ranging from 8 to 190 nm), which were obtained from nonliving surface-initiated polymerization reactions.^45−47^ Interestingly, p(FAcr) layers were visible to the naked eye as an intensively colored film (see Figure 8). The coloring results from Fabry–Perot interference, which is determined by the thickness and the refractive index of a thin layer meeting Bragg’s condition. Interference coloring appears when a layer with a thickness of around 100 nm is deposited on a highly reflective surface. This phenomenon was, for example, used by Kado et al., who produced three-dimensional polymeric nanostructures, which displayed interference coloring in dependence of their film thickness.^48^

### Photopatterning

Besides polymerization all over the wafer surface, spatially resolved photopolymerization reactions were carried out with PFAcr on functionalized silicon wafers. In a first step, the initiating species was locally deactivated by illumination through a photomask. Subsequently, the photomask was removed, the substrates were completely covered with PFAcr, and a flood illumination (1 min) was carried out, allowing for surface-initiated photopolymerization only at previously shielded and thus still photoactive areas. After rinsing, the patterned polymer layer of p(PFAcr) was investigated by optical microscopy as depicted in Figure 8, which shows a well-reproduced logo with clear boundaries. Both the zones bearing a photografted polymer layer and the zones lacking a graft layer were characterized with XPS measurements. The resulting survey spectra are included in Figure 8. In accordance with the molecular structure of p(PFAcr), the polymer layer consists of C, O, and above all F (Figure 8, red curve). Notably, very small amounts of F (690 eV) were also found on the surface of the nonpolymerized areas (compare black curve in Figure 8), which can be assigned to a few grafted chains of p(PFAcr). This is supported by the corresponding high-resolution XPS spectra of C1s and F1s (see Figure S9). From the deconvolution procedure applied to C1s, five contributions can be identified, two of which clearly proving the presence of CF_2_ (291.4 eV) and CF_3_ (293.8 eV) units.^38^ Moreover, the F1s spectrum shows only one peak at 688–689 eV, which is attributed to organic fluorine compounds. We assume that, after the deactivation step (illumination without monomer being present), some few photosensitive moieties still remained on the surface, which in turn were capable to initiate photopolymerization of PFAcr during the second (flood) illumination step. Those moieties could be either (a) Ge-COMes formed via recombination (see Scheme 1) or (b) a germy-peroxyl species derived from the reaction between the germyl radical and oxygen from the air followed by hydroperoxide formation.^49^

**Figure 9:**
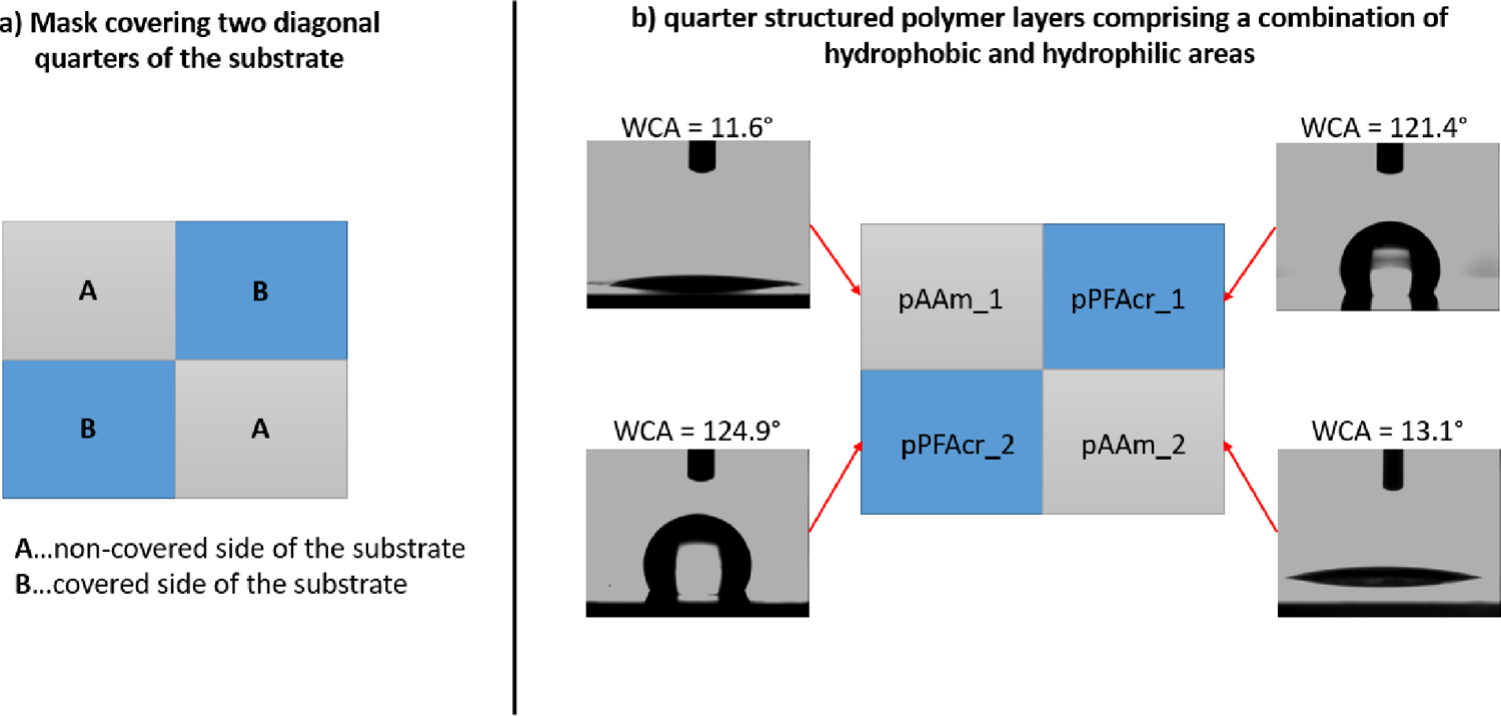
(a) Schematic representation of the applied photomask to prepare quarter structured polymer layers and (b) WCA measurements on the combined polymer surface comprising both hydrophobic and hydrophilic areas.

In addition, combined polymer layers with both p(PFAcr) and p(AAm) regions were prepared by the use of a simple designed photomask. Therefore, a solution of AAm in EtOH was drop coated onto a Si wafer functionalized with photoinitiator **2** and illuminated through a photomask that covers two-quarters of the wafer diagonal to each other (see Figure 9a). After light exposure through the respective photomask, the wafers were washed with EtOH, drop coated with (PFAcr), and again illuminated without a mask to initiate graft polymerization in those regions that were covered in the first step. After thorough rinsing and drying, XPS and contact angle measurements were performed.

As shown in Figure 9b, water contact angle measurements of the quarter structured polymer layer indeed proved hydrophilic areas of p(AAm) (WCA < 15°) in those areas where the photografting was performed in the first step, whereas hydrophobic areas of p(PFAcr) (WCA > 120°) were found in those areas that were initially shielded by the mask and covered with a photografted polymer layer in the second reaction step.

**Figure 10:**
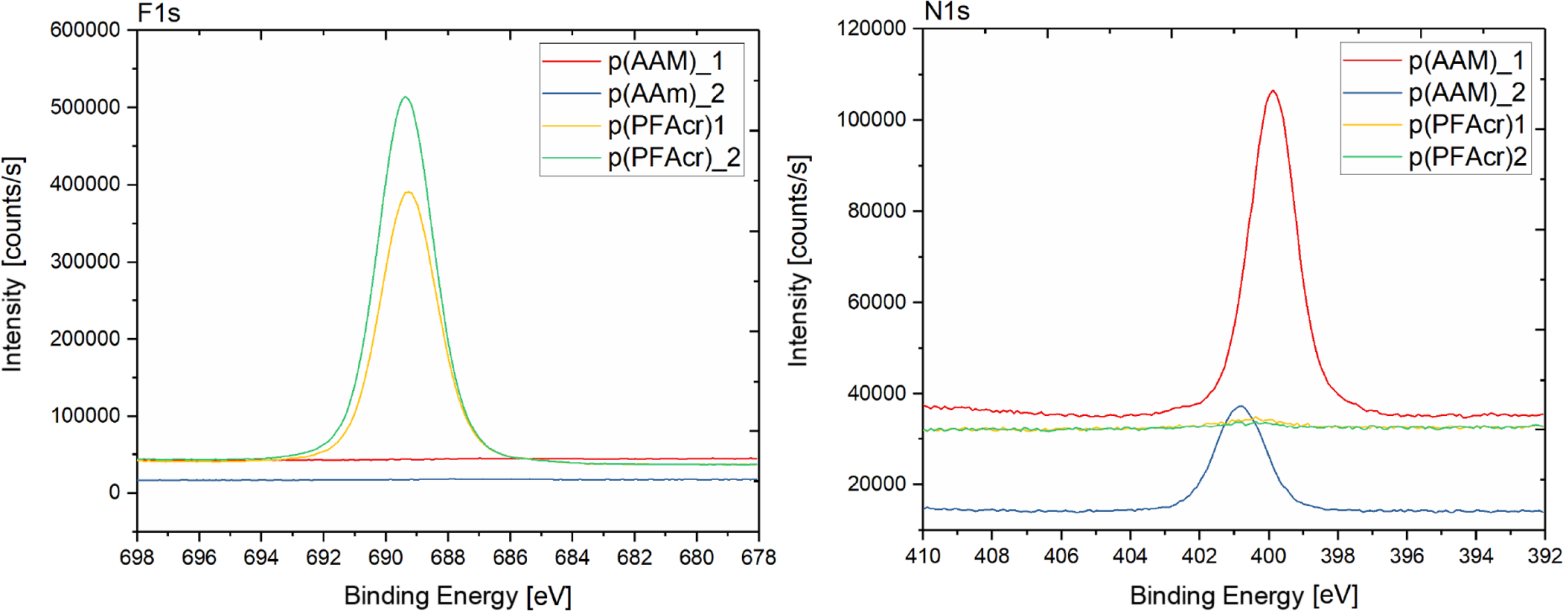
XPS high-resolution spectra of F1s and N1s on the quarter structured wafer bearing photografted layers of p(PFAcr) and p(AAm).

These results were confirmed by XPS measurements, in which one measurement was done in each quarter of the wafer with the combined polymer layers of p(PFAcr) and p(AAm). The quantitative evaluation of the survey spectra (see Table 5) revealed successful surface initiated polymerization in both steps. The C content increased, whereas the Si and Ge content decreased significantly, in all investigated areas when compared to the surface of a Si wafer modified with compound **2** (without photografting). Moreover, the regions of p(PFAcr) and p(AAm) can be clearly distinguished by the respective F and N contents according to the chemical structure of the applied monomers. Whereas the p(PFAcr) areas were characterized by high F contents of up to 40 at. %, no F was found for the p(AAm) regions. Contrariwise, significant amounts of N (up to 12 at. %) were only found in the quarters covered with p(AAm). In addition, these findings are visualized by the comparison of the respective high-resolution spectra of F1s and N1s in Figure 10.

**Table 5:** Overview of Atomic Surface Compositions Obtained from XPS Survey Spectra of a Neat Si Wafer, a Si Wafer Functionalized with a Photoinitiator, and the Quarter Structured Wafer Bearing Photografted Layers of P(PFAcr) and P(AAm)^a^

	chemical composition [at. %] on quarter structured wafer								
	C	Ge	F	O	Si	Cl	N	Na	Ca
neat Si	14.7 ± 0.6			32.3 ± 0.3	51.6 ± 0.0	1.3^b^	0.9 ± 0.1		
Si wafer functionalized with photoinitiator	10.4 ± 0.5	2.5 ± 0.1	2.9 ± 0.1	49.8 ± 0.3	33.4 ± 0.2			0.9 ± 0.0	
p(AAm) 1	39.6			33.4	17.4		9.1	0.5	
p(AAm) 2	50.1			25.6	11.7		11.5	0.5	0.5
p(PFAcr) 1	32.23		40.1	17.6	10.0				
p(PFAcr) 2	28.9	0.8	32.2	23.2	14.38		0.5		

a This table contains mean values for the neat Si wafer and the wafer modified with compound **2**, whereas for the spots measured on the quarter structured wafer, single values are reported because of the small dimensions of the individual area.

b Single value, detected on only one of 4 measurement spots on the sample.

## Conclusions

Herein, we report the straightforward synthesis of a novel germanium-based photoinitiator bearing a bromine moiety (bromo-tris(2,4,6-trimethylbenzoyl)germane **2**), which can easily be coupled to hydroxyl-rich surfaces as is the case for (oxygen-plasma activated) quartz plates and silicon wafer substrates. Our novel synthetic approach is based on a metal-halide exchange reaction of the triacylgermenolate with 1,2-dibromoethane. This strategy circumvents the usage of toxic mercury(II) chloride, which represents the state-of-the-art protocol toward halogen substituted acylgermanes. Compound **2** was isolated in good yields and characterized by means of NMR, UV–vis spectroscopy, as well as single-crystal X-ray crystallography.

The coupling procedure of compound **2** onto silicon wafer and quartz plate surfaces was carried out by immersing the previously activated substrates into a THF solution of compound **2** and DIPEA at elevated temperatures. As evidenced by XPS, the presence of germanium and the absence of bromine on the modified substrates proved the covalent coupling of compound **2**.

Furthermore, it is shown that the immobilized Ge-based photoinitiator is capable of efficiently initiating a visible-light-induced and surface-mediated polymerization reaction of various functional monomers. The resulting layers were characterized with regard to their chemical composition employing XPS, UV–vis, and fluorescence spectroscopy as well as water contact angle measurements to investigate their specific surface functionality. Using neat 1*H*,1*H*,2*H*,2*H*-perfluorodecyl acrylate as the monomer for the “grafting from” reaction, thick hydrophobic layers (thickness up to 126 nm) with water contact angles >120 °C were obtained. Also, those layers were visible to the naked eye because of the strong coloring of the surface based on an interference effect. When performing a spatially resolved depletion of the immobilized photoinitiator, patterned photografted polymer layers are obtained after flood illumination in the presence of monomers. The ability of those patterned layers to withstand multiple rinsing cycles without detectable loss of optical resolution once more proved their covalent attachment to the surface. In addition, acrylamide was used for surface-initiated polymerization reactions, leading to pronounced hydrophilic surfaces as demonstrated by a water contact angle <15°. With a similar approach, structured surfaces bearing both hydrophobic and hydrophilic areas were prepared. Furthermore, poly(fluorescein-*o*-acrylate) and poly(1-pyrenemethyl methacrylate) were successfully grafted from quartz plates bearing the immobilized photoinitiator **2**.

Summing up, the “grafting from” method reported here provides an attractive possibility to modify and functionalize silicon surfaces, which could be also applied for a wide range of materials carrying, for example, hydroxyl, amine, or other Lewis bases at the surface. Considering the advantages of germanium-based photoinitiators, such as nontoxicity, visible-light absorbance up to 480 nm, and high light sensitivity, this may open up new perspectives for surface modifications, especially in the field of biomedicine.

## Supporting Information Available

Crystallographic information (CIF)
NMR spectroscopy, UV–vis spectroscopy, X-ray crystallography, FTIR spectroscopy, and XPS results (PDF)

## Supplementary Material

am3c05528_si_001.cif

## Abbreviations

AAm: acrylamide
DCM: dichloromethane
DIPEA: *N*,*N*-diisopropylethylamine
EtOH: ethanol
FlAcr: fluorescein-*o*-acrylate
p(FlAcr): poly(fluorescein-*o*-acrylate)
HFIP: hexafluoroisopropanol
p(AAm): poly(acrylamide)
PFAcr: 1*H*,1*H*,2*H*,2*H*-perfluorodecyl acrylate
p(PFAcr): poly(1*H*,1*H*,2*H*,2*H*-perfluordecyl-acrylate)
PyrMAcr: 1-pyrenemethyl methacrylate
p(PyrMAcr): poly(1-pyrenemethyl methacrylate)
SI-ATRP: surface-initiated atom-transfer radical polymerization
SI-PET-RAFT: surface-initiated photoinduced electron/energy transfer reversible addition–fragmentation chain transfer polymerization
SI-PIMP: surface-initiated photoiniferter-mediated polymerization
THF: tetrahydrofurane
TMS: tetramethylsilane
WCA: water contact angle
XPS: X-ray photoelectron spectroscopy
