# Supplementary material for: Surface-Initiated Polymerizations Mediated by Novel Germanium-Based Photoinitiators
Source: ACS Appl Mater Interfaces. 2023 Jun 23;15(26):31836–48. doi: 10.1021/acsami.3c05528 (PMC10326805; doi:10.1021/acsami.3c05528)
Supplement: Supplementary file 2 — am3c05528_si_002.pdf [file am3c05528_si_002.pdf]

## Supporting Information

# Surface-initiated polymerizations mediated by novel Germanium-based Photoinitiators

Matthias Müller,[1] Manfred Drusgala,[2] Roland C. Fischer,[2] Ana Torvisco,[2] Wolfgang Kern,[1,3] Michael Haas[2*] and Christine Bandl[1*]

[1] Montanuniversität Leoben, Institute of Chemistry of Polymeric Materials; Otto-Glöckel-Strasse 2, A 8700 Leoben – Austria
[2] Graz University of Technology, Institute of Inorganic Chemistry; Stremayrgasse 9, A-8010 Graz– Austria

[3] Polymer Competence Center Leoben GmbH, Roseggerstrasse 12, A–8700 Leoben – Austria

*Christine Bandl[1] and Michael Haas[2]

[1] Montanuniversität Leoben, Institute of Chemistry of Polymeric Materials; Otto-Glöckel-Straße 2, A 8700 Leoben – Austria; Christine.bandl@unileoben.ac.at

[2] Graz University of Technology, Institute of Inorganic Chemistry; Stremayrgasse 9, A-8010 Graz– Austria; Michael.haas@tugraz.at

# Table of Content

NMR-Spectroscopy ........ 2
NMR spectra of 2 ........ 2
NMR spectra of 2a ........ 4
UV-Vis-Spectroscopy ........ 6
UV-Vis spectroscopy of 2 ........ 6
UV-Vis spectroscopy of 2a ........ 6
X-ray Crystallography ........ 7
FTIR-spectroscopy ........ 8
XPS Results ........ 9

## NMR-Spectroscopy

### NMR spectra of 2

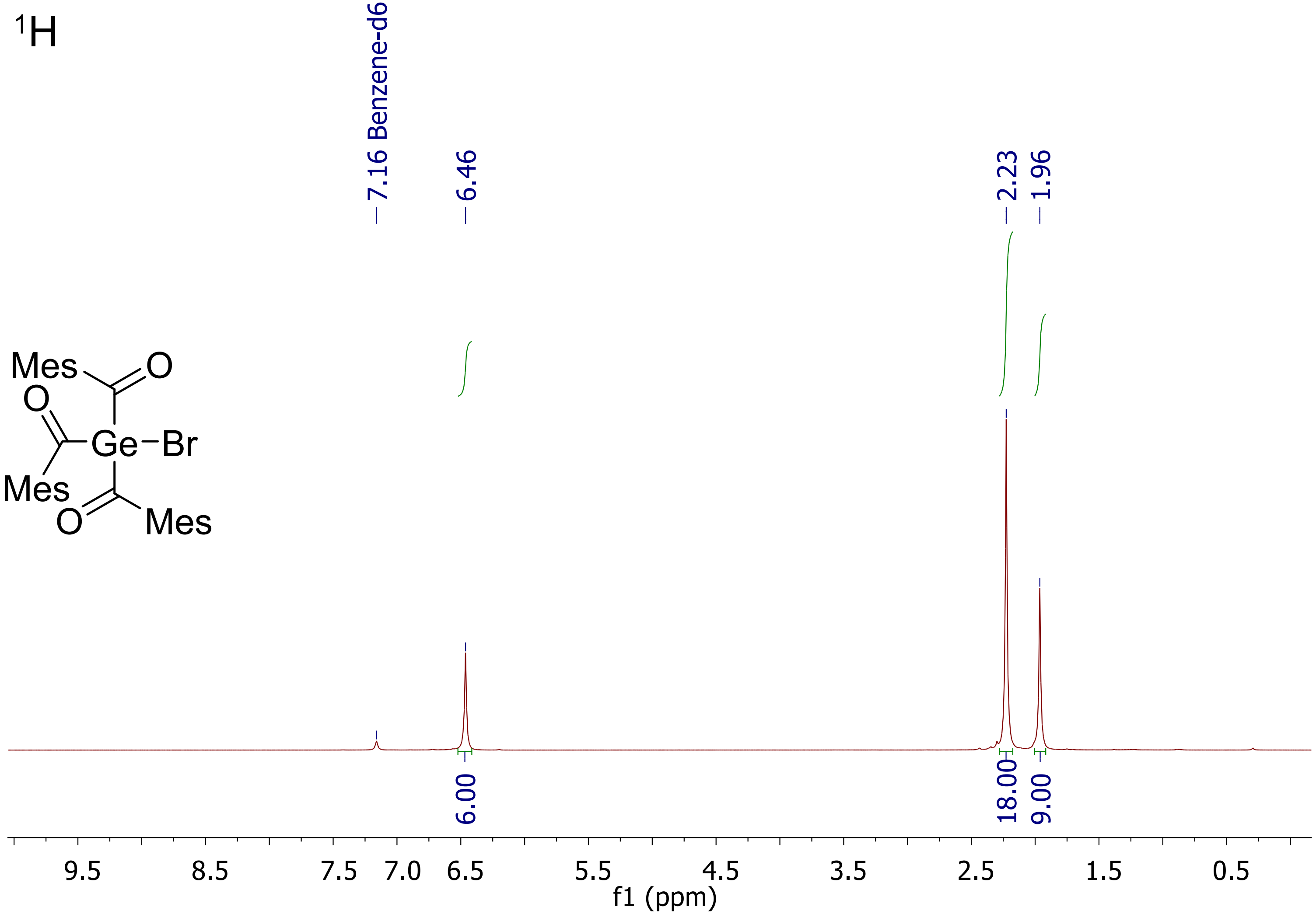


**Figure S1.** $^{1}H$- spectrum of **2** (benzene-$d_6$ solution, vs ext. TMS, ppm)

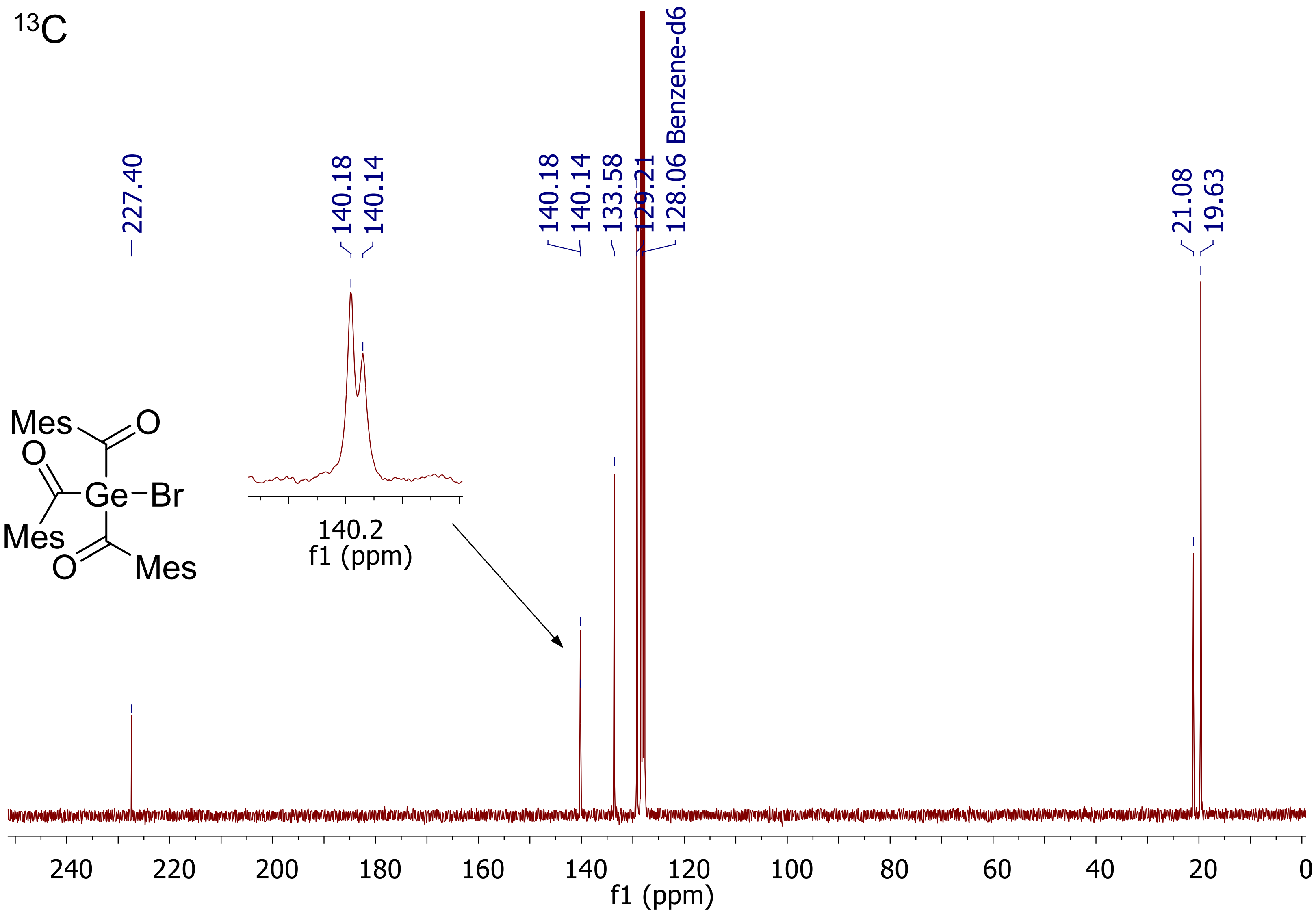


**Figure S2.** $^{13}$C- spectrum of **2** (benzene-$d_6$ solution, vs ext. TMS, ppm)

**NMR spectra of 2a**

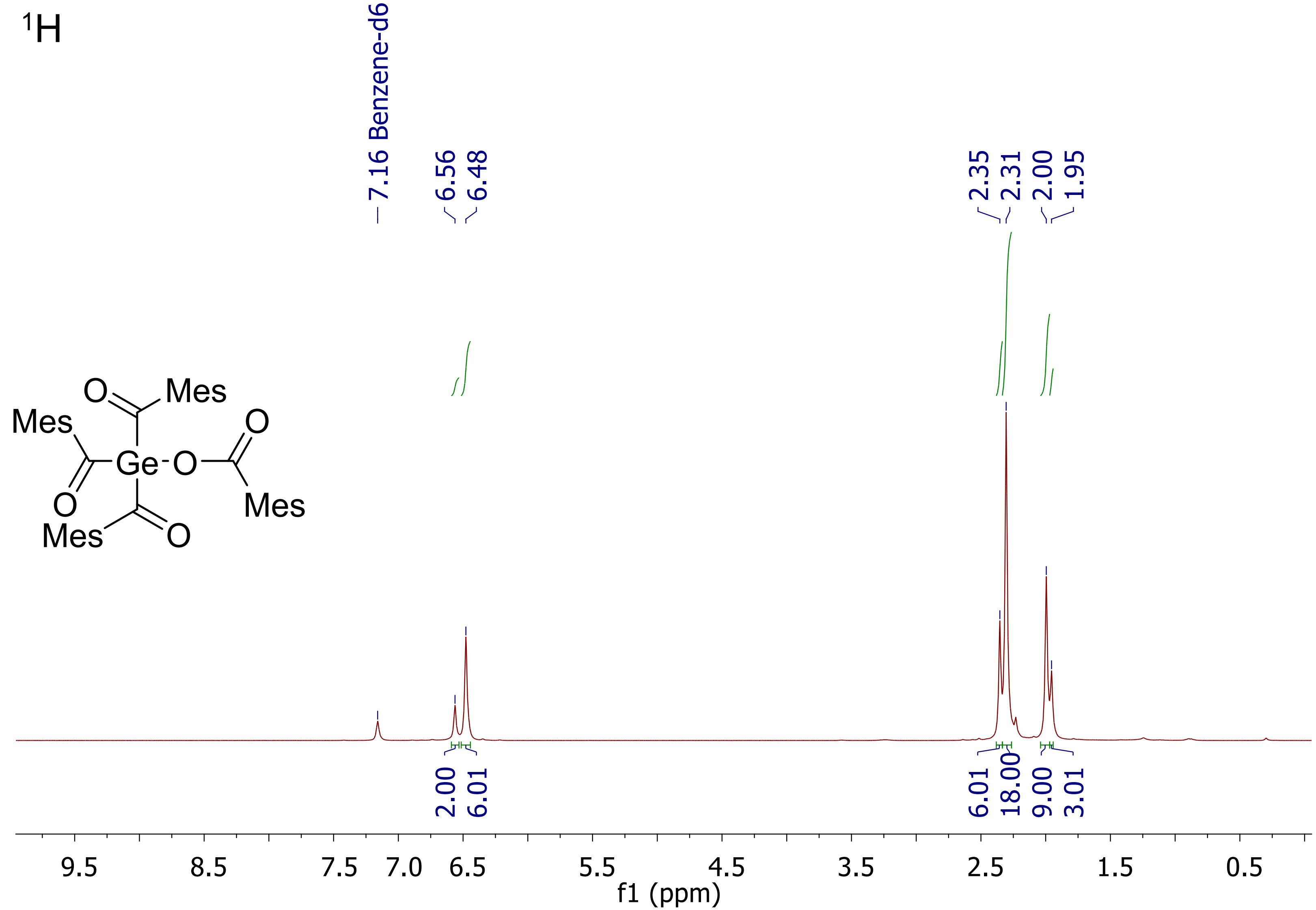


**Figure S3.** $^{1}H$- spectrum of **2a** (benzene-$d_6$ solution, vs ext. TMS, ppm)

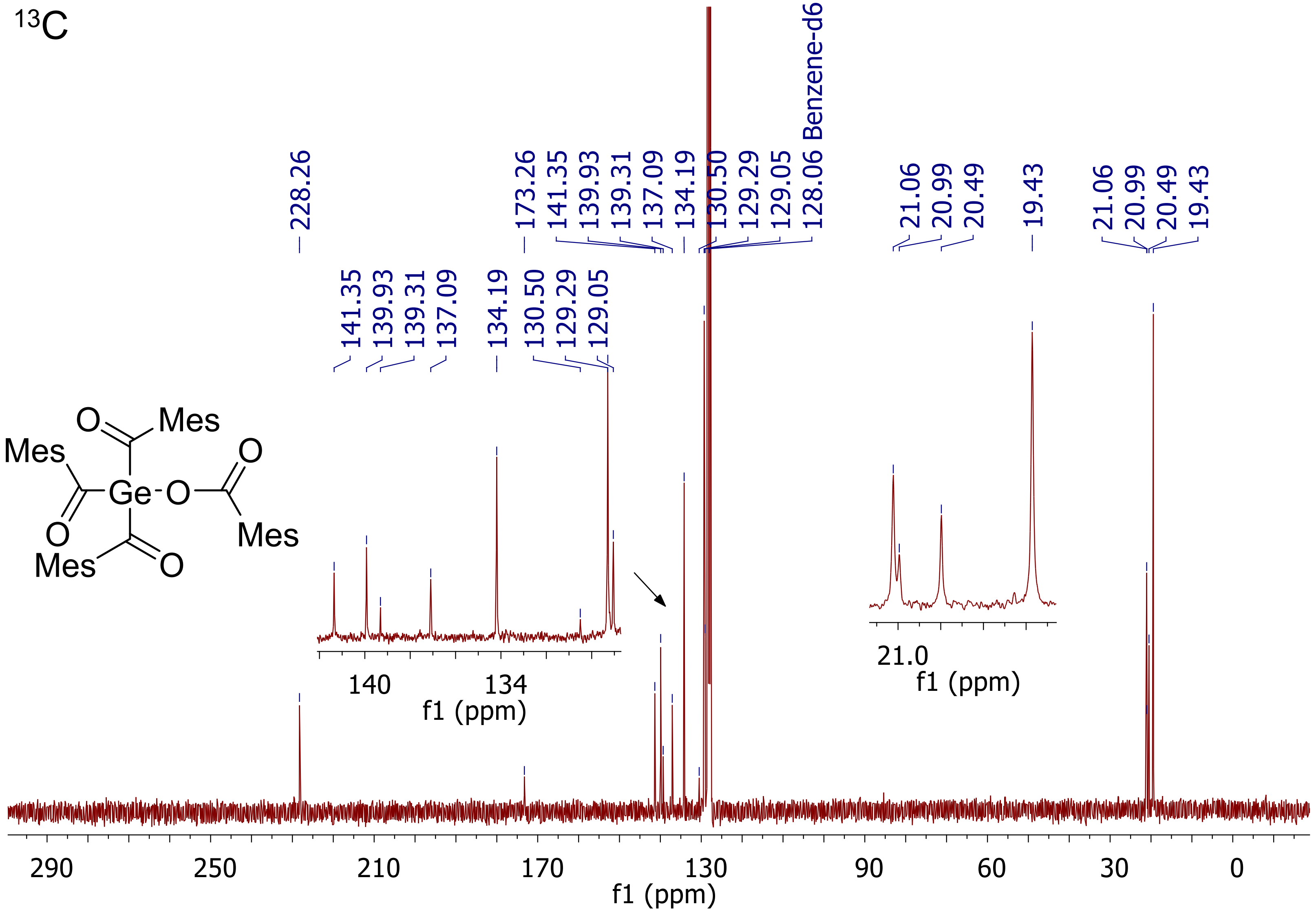


**Figure S4.** $^{13}$C- spectrum of **2a** (benzene-$d_6$ solution, vs ext. TMS, ppm)

## UV-Vis-Spectroscopy

### UV-Vis spectra of compound 2

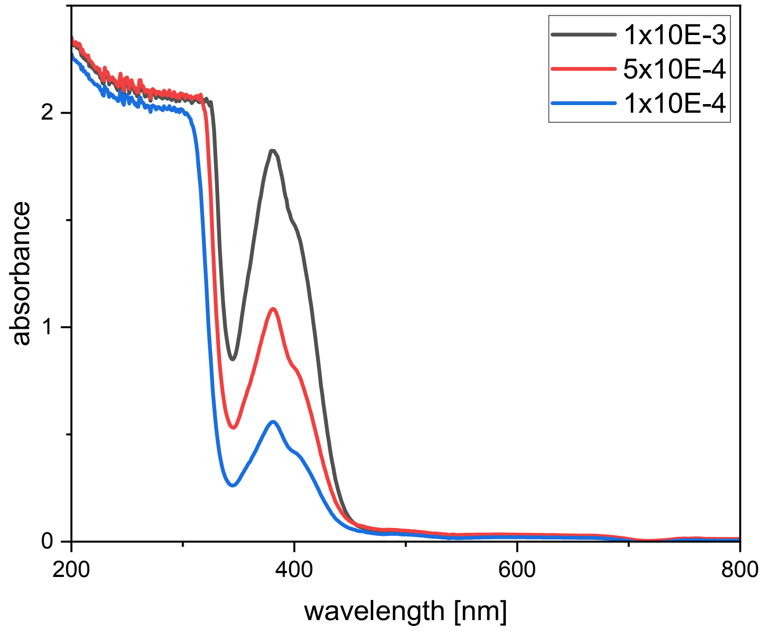


**Figure S5.** UV-Vis spectrum of **2** measured in THF at three different concentrations (M [mol· $l^{-1}$] = $1x10^{-3}$, $5x10^{-4}$, $1x10^{-4}$)

### UV-Vis spectroscopy of 2a

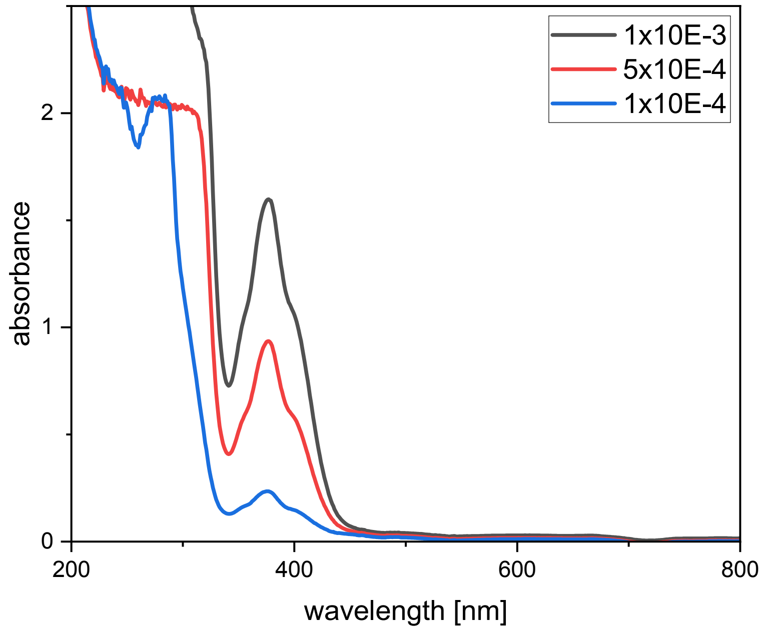


**Figure S6.** UV-Vis spectrum of **2** measured in THF at three different concentrations (M [mol· $l^{-1}$] = $1x10^{-3}$, $5x10^{-4}$, $1x10^{-4}$)

## X-ray Crystallography

All crystals suitable for single crystal X-ray diffractometry were removed from a vial or a Schlenk and immediately covered with a layer of silicone oil. A single crystal was selected, mounted on a glass rod on a copper pin, and placed in the cold $N_2$ stream provided by an Oxford Cryosystems cryostream. XRD data collection was performed for compounds **2** and **2a**, on a Bruker APEX II diffractometer[1] with use of an IμS microsource (Incoatec microfocus) sealed tube of Mo Kα radiation (λ= 0.71073 Å) and a CCD area detector. Data integration was carried out using SAINT.[1] Empirical absorption corrections were applied using SADABS.[2] The structures were solved with use of the intrinsic phasing option in SHELXT[3] and refined by the full-matrix least-squares procedures in SHELXL[4] as implemented in the program SHELXLE.[5] The space group assignments and structural solutions were evaluated using PLATON.[6] Non-hydrogen atoms were refined anisotropically. Hydrogen atoms were located in calculated positions corresponding to standard bond lengths and angles. CIF files were edited, validated and formatted either with the programs encifer, publCIF, or Olex2. CCDC 2233933-2233934 contain the supplementary crystallographic data for compounds **2** and **2a** respectively. These data can be obtained free of charge from The Cambridge Crystallographic Data Centre via www.ccdc.cam.ac.uk/data_request/cif. Table S1 contains crystallographic data and details of measurements and refinement for compounds **2** and **2a**.

**Table S1.** Crystallographic data and details of measurements for compounds **2** and **2a**. Mo Kα (λ=0.71073Å). R1= Σ/ $|F_o|$- $|F_c|/|\Sigma|F_d$; wR2 = $[\Sigma_w(F_o^2\text{-}F_2^2)^2/\Sigma_w(F_o^2)^2]^{1/2}$

| **Compound** | **2** | **2a** |
|---|---|---|
| Formula | $C_{30}H_{33}BrGeO_3$ | $C_{40}H_{44}GeO_5$ |
| Fw (g mol$^{-1}$) | 594.06 | 677.34 |
| *a* (Å) | 11.7256(7) | 10.3676(7) |
| *b* (Å) | 16.9232(9) | 12.3101(8) |
| *c* (Å) | 14.6024(10) | 27.0059(16) |
| *α* (°) | 90 | 90 |
| *β* (°) | 107.624(5) | 97.131(2) |
| *γ* (°) | 90 | 90 |
| *V* (Å$^3$) | 2761.6(3) | 3420.0(4) |
| *Z* | 4 | 4 |
| Crystal size (mm) | 0.25 × 0.14 × 0.09 | 0.21 × 0.09 × 0.07 |
| Crystal habit | Block, yellow | Block, yellow |
| Crystal system | Monoclinic | Monoclinic |
| Space group | $P2_1/c$ | $P2_1/n$ |
| $d_{calc}$ ( Mg m$^{-3}$) | 1.429 | 1.315 |
| $\mu$ (mm$^{-1}$) | 2.58 | 0.939 |
| *T* (K) | 100(2) | 100 |
| 2$\theta$ range (°) | 2.2–29.4 | 2.031–28.035 |
| *F*(000) | 1216 | 1424 |
| $T_{min}$, $T_{max}$ | 0.504, 0.747 | 0.569, 0.746 |
| $R_{int}$ | 0.100 | 0.058 |
| No. of measured, independent and observed [$I > 2\sigma(I)$] reflections | 221882, 5437, 4840 | 29435, |
| independent reflections | 5437 | 8200 |

| | | |
|---|---|---|
| No. of parameters, restraints | 325, 0 | 427, 0 |
| Δρmax, Δρmin(e Å$^{-3}$) | 0.38, -0.35 | 0.81, -0.53 |
| R1, wR2 (all data) | R1 = 0.0313<br>wR2 = 0.0648 | R1 = 0.0606<br>wR2 = 0.0980 |
| R1, wR2 (>2$\sigma$) | R1 = 0.0236<br>wR2 = 0.0575 | R1 = 0.0430<br>wR2 = 0.0922 |

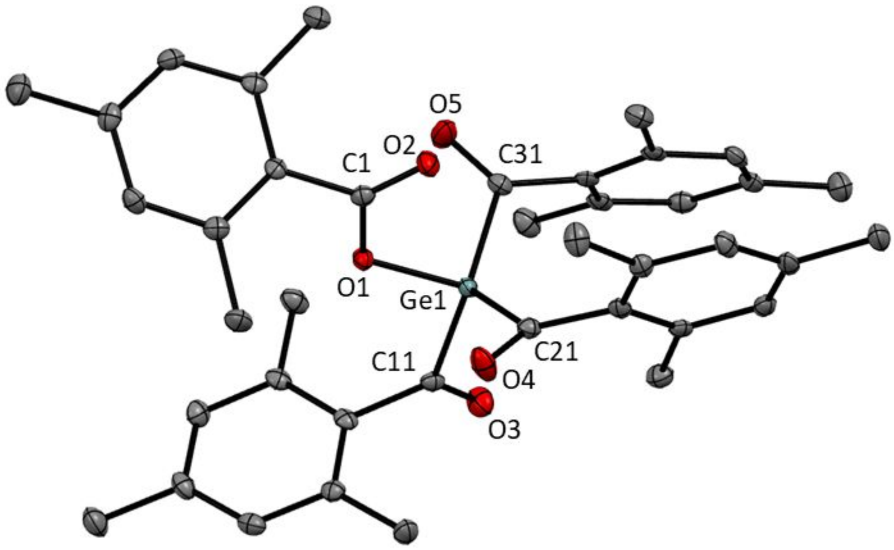


**Figure S7.** ORTEP representation of compound **2a**

## FTIR-spectroscopy

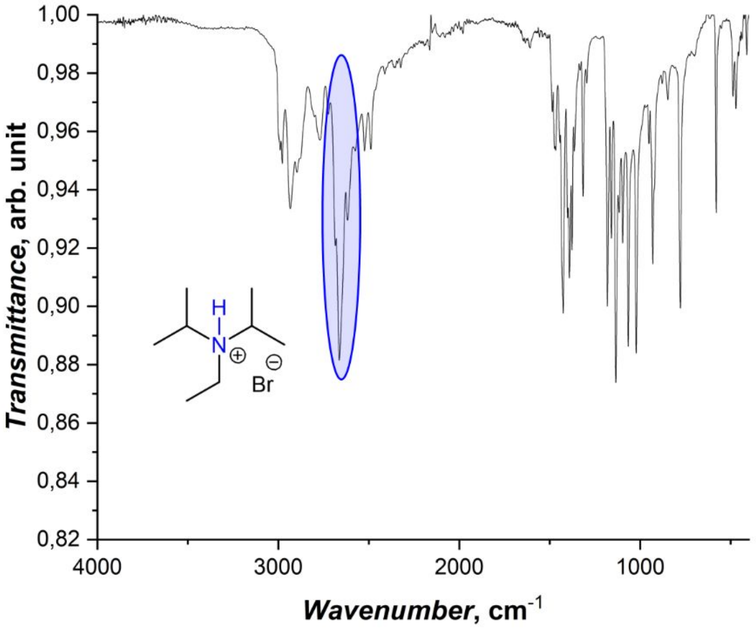


**Figure S8.** FTIR spectra of N-ethyl,N-isopropyl propan-2-ammonium bromide

## XPS Results

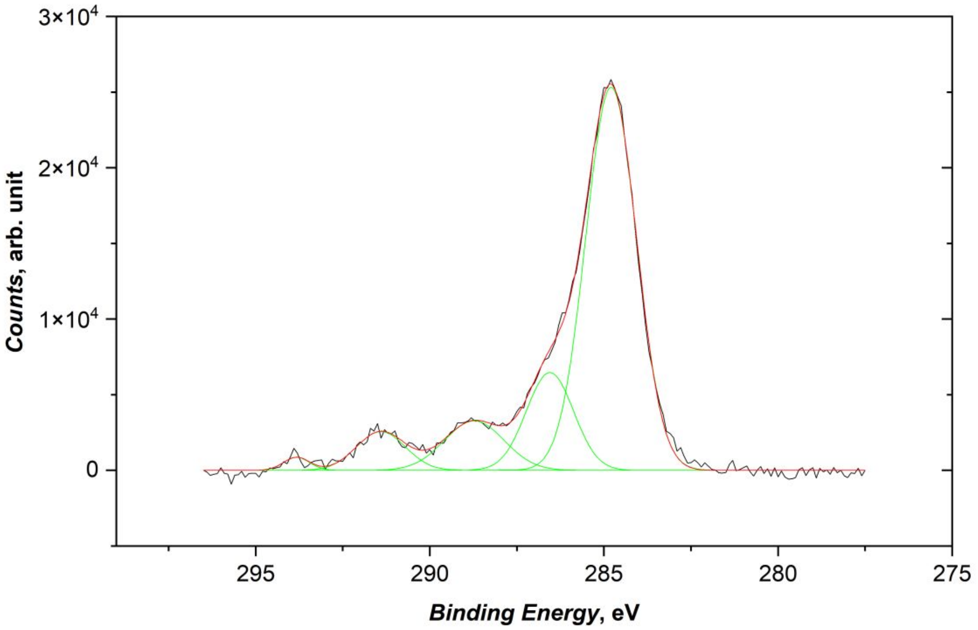


**Figure S9.** XPS C1s-deconvolution spectra of patterned samples (non-polymerized area)

**References:**


[1] Bruker APEX2 and SAINT, Bruker AXS Inc.: Madison, Wisconsin, USA, 2012.

[2] a) Sheldrick, G. M. SHELXS97, Univ. Göttingen, Ger. , 1997.; b) R. H. Blessing, *Acta crystallographica. Section A, Foundations of crystallography* **1995**, *51 ( Pt 1)*, 33.

[3] G. M. Sheldrick, *Acta crystallographica. Section A, Foundations and advances* **2015**, *71*, 3.

[4] G. M. Sheldrick, *Acta crystallographica. Section C, Structural chemistry* **2015**, *71*, 3.

[5] C. B. Hübschle, G. M. Sheldrick, B. Dittrich, *Journal of applied crystallography* **2011**, *44*, 1281.

[6] a) A. L. Spek, *Journal of applied crystallography* **2003**, *36*, 7; b) A. L. Spek, *Acta crystallographica. Section D, Biological crystallography* **2009**, *65*, 148.
